# Multiple bHLH/MYB-based protein complexes regulate proanthocyanidin biosynthesis in the herbage of *Lotus* spp.

**DOI:** 10.1007/s00425-023-04281-2

**Published:** 2023-12-02

**Authors:** Francisco José Escaray, Maria Cristina Valeri, Francesco Damiani, Oscar Adolfo Ruiz, Pedro Carrasco, Francesco Paolocci

**Affiliations:** 1 Instituto de Biología Molecular de Plantas (IBMCP) Universitat Politécnica de València – C.S.I.C, Ciudad Politécnica de la Innovación, Edificio 8E, Ingeniero Fausto Elio, s/n, 46022 Valencia, Spain; 2 Institute of Biosciences and BioResources (IBBR), Consiglio Nazionale Delle Ricerche, Via Madonna Alta, 130, 06128 Perugia, Italy; 3 Biotecmed, Department of Biochemistry and Molecular Biology, University of València, 46100 Burjassot, Valencia Spain; 4 Unidad de Biotecnología 1, Instituto Tecnológico de Chascomús (INTECh), Consejo Nacional de Investigaciones Científicas y Técnicas (CONICET), Avenida Intendente Marino KM 8.2, 7130 Chascomús, Buenos Aires Argentina

**Keywords:** Condensed tannins, Forage breeding, Protein, Protein interaction, Ruminal bloating, Transcriptome, Transcription factor

## Abstract

**Main conclusion:**

**The complexes involving MYBPA2, TT2b, and TT8 proteins are the critical regulators of** ***ANR*** **and** ***LAR*** **genes to promote the biosynthesis of proanthocyanidins in the leaves of** ***Lotus*** **spp.**

**Abstract:**

The environmental impact and health of ruminants fed with forage legumes depend on the herbage's concentration and structure of proanthocyanidins (PAs). Unfortunately, the primary forage legumes (alfalfa and clover) do not contain substantial levels of PAs. No significant progress has been made to induce PAs to agronomically valuable levels in their edible organs by biotechnological approaches thus far. Building this trait requires a profound knowledge of PA regulators and their interplay in species naturally committed to accumulating these metabolites in the target organs. Against this background, we compared the shoot transcriptomes of two inter-fertile *Lotu*s species, namely *Lotus tenuis* and *Lotus corniculatus*, polymorphic for this trait, to search for differentially expressed *MYB* and *bHLH* genes. We then tested the expression of the above-reported regulators in *L. tenuis* x *L. corniculatus* interspecific hybrids, several *Lotus* spp., and different *L. corniculatus* organs with contrasting PA levels. We identified a novel MYB activator and MYB-bHLH-based complexes that, when expressed in *Nicotiana benthamiana,* trans-activated the promoters of *L. corniculatus anthocyanidin reductase* and *leucoanthocyanidin reductase 1* genes. The last are the two critical structural genes for the biosynthesis of PAs in *Lotus* spp. Competition between MYB activators for the transactivation of these promoters also emerged. Overall, by employing *Lotus* as a model genus, we refined the transcriptional network underlying PA biosynthesis in the herbage of legumes. These findings are crucial to engineering this trait in pasture legumes.

**Supplementary Information:**

The online version contains supplementary material available at 10.1007/s00425-023-04281-2.

## Introduction

Legumes (Fabaceae) are critical components of natural and agricultural ecosystems and are the primary source of plant protein for human and livestock nutrition. Pasture legumes' nutritional value must be improved to meet the rising world's demand for cheap and safe livestock food products and genuinely sustainable livestock farming (Lüscher et al. 2014; Notenbaert et al. 2021). Proanthocyanidins (PAs), known as condensed tannins, are polymeric flavonoids that significantly affect legume quality (Mueller-Harvey et al. 2019). By binding dietary proteins, PAs slow down their fermentation in the rumen and, in turn, increase the conversion rate of plant proteins into animal proteins while decreasing ruminal bloating and the emission of greenhouse gases in the atmosphere (Aerts et al. 1999; Hess et al. 2006; Patra and Saxena 2010). PAs also exert an anti-parasitic effect against ruminant and non-ruminant gastrointestinal parasites. Still, when their concentration is too high, they reduce the voluntary intake by the animals and lower the nutritional value of forage diets (Mueller-Harvey et al. 2019). Unfortunately, only a few forage legumes of temperate climates synthesize these metabolites in their edible herbage; most do it in the seed coat (Paolocci et al. 2007). Thus, understanding the genetic control of PAs is vital to engineer the biosynthesis of these metabolites in the herbage of the most valuable forage legumes (such as *Medicago* and *Trifolium* spp., *Lotus tenuis*), currently one of the primary goals of forage breeders worldwide.

The PA biosynthetic pathway has been characterized in many species. The building blocks of these polymers are the flavan-3-ols epicatechins and catechins that are synthesized by the reduction of anthocyanidins and leucoanthocyanidins via anthocyanidin reductase (ANR) and leucoanthocyanidin reductase (LAR), respectively (Tian et al. 2008). More recently, it has also been shown that the biosynthetic routes to epicatechin starter and extension units can differ and that LAR plays a crucial role in producing epicatechin starter units (reviewed in Lu et al. 2022). This additional role of LAR explains the higher levels of epicatechin rather than catechin units found in species overexpressing functional LARs (Liu et al. 2013).

The regulation of flavonoid genes occurs mainly at the transcriptional level. Members of the R2R3-MYB, the basic helix-loop-helix (bHLH), and WD-repeat families (Davies and Schwinn 2003; Lepiniec et al. 2006) form the MYB-bHLH-WDR (MBW) complex that regulates the organ- and tissue-specific expression of the different branches of the flavonoid pathway (Davies and Schwinn 2003; Lepiniec et al. 2006; Dubos et al. 2010; Xu et al. 2015; Lafferty et al. 2022). The MYB components provide branch specificity to this complex (Broun 2005). In Arabidopsis, AtMYB123/TT2 is the R2R3-MYB that targets the MBW complex to the PA pathway (Nesi et al. 2001, 2002). Orthologue genes of *AtMYB123* have been characterized in *Lotus japonicus* (Yoshida et al. 2010b), *Medicago* spp. (Verdier et al. 2012), and *Trifolium* spp. (Hancock et al. 2012). Furthermore, other MYB proteins related to transcriptional activation of PA biosynthesis have been described: MYB5 from *Medicago truncatula* (Liu et al. 2014), MYBPA1 and MYBPA2 from *Vitis vinifera* (Bogs et al. 2007) or MYB7 from *Prunus persica* (Terrier et al. 2008). MYBs, like MYB134 from *Trifolium repens* and MYB1 from *Fragaria* x *ananassa*, have been shown to act as repressors of PA-genes in legumes (Albert 2015; Paolocci et al. 2011). These metabolites accumulate in an organ-specific manner that depends upon a finely tuned balance between activator and repressor proteins (Ma and Constabel 2019; Zho et al. 2019).

Despite the wealth of knowledge on PA biosynthesis in legume and non-legume species, no significant steps forward have been made in inducing PAs to agronomical valuable levels in the herbage of the most important forage legumes by trans-genetics (Zhou et al. 2015). Nevertheless, we note that most of the genes employed for this purpose are from species that do not naturally accumulate PAs in the herbage, such as *L. japonicus*, *M. truncatula*, and Arabidopsis (Li et al. 1996; Debeaujon et al. 2003). Thus, they might operate within complexes specific to reproductive rather than vegetative organs. Only very recently, levels of foliar PAs sufficient to reduce ammonia and methane production in the rumen in vitro assays were reached in *Trifolium repens*, provided that the exogenous *TaMYB14-1* transcription factor (TF) was expressed in recipient germplasm already committed to synthesizing anthocyanins in leaves (Roldan et al. 2022). To search for PA-specific TFs regulating the synthesis of these metabolites in the foliage of agronomically important forage species, here we exploited the genetic variability of two *Lotus* spp. polymorphic for the PA trait, namely *L. tenuis* and *Lotus corniculatus*. Being inter-fertile and unable to accumulate anthocyanins in their leaf blades and shoot apexes, these two species offer the advantage of assessing the levels of PAs and those of structural and regulatory genes of this pathway in their progeny and anthocyanin-free germplasm contexts (Robbins et al. 2003; Escaray et al. 2014; Aoki et al. 2021). The latter point is crucial in light that PA and anthocyanin genes can be co-regulated through the same core MBW regulatory complex (Yue et al. 2023), somewhat hampering the identification of PA-specific regulators from those controlling multiple branches of the flavonoid pathway. Thus, the transcriptomes of the shoot apexes of a cultivar of *L. tenuis* grown in South America and with negligible levels of PAs (Escaray et al. 2012) and that of a wild, diploid genotype of *Lotus corniculatus*, which accumulates high levels of PAs throughout the leaf mesophyll and in the stems (Escaray et al. 2014) have been compared and differentially expressed (DE) *MBW* components retrieved. Then, their expression was assessed in leaves of *L. tenuis x L. corniculatus* interspecific hybrids, which showed intermediate levels of PAs compared to the two parents and F2 progeny with contrasting levels of leaf PAs (Escaray et al. 2014, 2017). Additionally, the expression of the *MBW* components retrieved above has been investigated in *Lotus* species and different *L. corniculatus* organs with varying levels of PAs. In essence, to avoid any possible interference or artifact due to ectopic gene expression, while taking into consideration the potential effects of environmental cues, the expression of the candidate PA regulators has been investigated in genome contexts that did not experience an ectopic expression of any given regulators and were grown under different environmental conditions. Finally, the candidates whose expression correlated with the levels of PAs in any comparison made, regardless of plant-growing conditions, were evaluated to form in vitro stable MBW complexes and induce the transcription of *Lotus ANR* and *LAR1* promoters. Overall, new actors and various MBW complexes with different relevance in controlling PA accumulation in the herbage of legumes have been characterized. Present findings are crucial to breeding bloat-safe forage legumes.

## Materials and methods

### Plant materials and cDNA sample sets

The *Lotus* spp. plant material employed in the present study was categorized as “PA-rich” and “PA-poor” genotypes according to the levels of PAs in their herbage. “PA-rich” genotypes showed more than 4 mg PA/g DW and were characterized by PA-accumulating cells throughout the leaf mesophyll. Conversely, in the “PA-poor” genotypes, the PA-accumulating cells were observed around the vascular tissues of only some of them, and the levels of PAs in these species were consistently lower than 1.5 mg/g DW (Escaray et al. 2014).

For RNAseq analysis, “PA-rich” and “PA-poor” apical shoots were collected from the diploid *L. corniculatus* accession “Charlii” and *L. tenuis* commercial cultivar “Pampa INTA”, respectively (Escaray et al. 2014). Fig. S1 provides information on RNA isolation and plant growing conditions of this material. The figure also gives information on the origin and features of the four sample sets employed to validate the involvement of PA biosynthesis of the regulatory genes selected after RNA-seq analysis. This was tested by investigating the correlation of their expression with PA levels and the expression profiles of main structural genes of this pathway in *Lotus* spp. accessions (sample sets 1–3) and *L. corniculatus* organs (set 4) polymorphic for this trait.

### RNA-seq analysis and functional annotation of transcripts

Samples for RNA-seq analysis (*n* = 3) were prepared as described in the TruSeq^®^ RNA Sample Preparation Guide (Illumina). High-performance, paired-end (2 × 100 bp) sequencing was performed on an Illumina Hiseq 1500 apparatus by the Institute of Agrobiotechnology of Rosario (Rosario, Argentina). Low-quality RNA-Seq reads (QScore < Q30) detected using FastQC (Version 0.11.2) were discarded (Andrews 2010). De novo assembly was performed by merging the high-quality reads using Trinity software (Grabherr et al. 2011) with a minimum contig length of 200 bases and a k-mer size of 25 bp. Functional annotation of assembled transcripts was performed by homology search (BLAST/Uniprot and SwissProt), protein domain identification (HMMER/PFAM), protein signal peptide and transmembrane domain prediction (signalP/tmHMM), and annotation databases search (eggnog/GO/Kegg) using Trinotate pipeline (Bryant et al. 2017).

The reads' FPKM (Fragments Per Kilobase Million) value was determined by the eXpress abundance estimation method (Roberts and Pachter 2013). Fold change for selected transcripts was estimated by FPKM of *L. corniculatus* / FPKM of *L. tenuis*.

### Selection and cloning of candidate MBW regulators

As reported above, all predicted proteins annotated as MYBs were selected. In addition, the two transcriptomes underwent a local Position-Specific Iterated BLAST (PSI-BLAST) using as queries the reference MYB proteins reported in Table S1. Likewise, this last strategy was used to identify possible bHLH and WDR members of the PA MBW regulatory complex.

Primer pairs (listed in Table S2) for each candidate regulator were designed to amplify their ORF from both species by RT-PCR and verify their sequence by Sanger. Neighbor-joining trees for each protein family were built using Mega 7 (Kumar et al. 2016).

### Gene expression analysis by qRT-PCR

The relative expression of PA structural and regulatory genes was verified on different sample sets by qRT-PCR, as reported by Escaray et al. (2017). The genes and relative primer pairs investigated by qRT-PCR are given in Table S2. The relative gene expression analysis was performed according to Pfaffl et al. (2002). The correlation analysis was performed using the Pearson test. All statistical analysis was performed using the Infostat program (Di Rienzo et al. 2011).

### Proanthocyanidin determination

An aliquot of samples ground for RNA isolation for RNA-seq analysis and for synthesizing the third and fourth sets of cDNAs was retained for PA determination performed as described in Escaray et al. (2012).

### Yeast-two-hybrid assay

Protein–protein interactions were evaluated by Y2H assay. The full CDS of *L. corniculatus TT8*, *TT2b*, *MYBPA2,* and *MYB5,* along with the control genes that code for MYBPA1 from grape and Sn (bHLH) from maize were fused either to the Gal4-DBD in the pDEST32 vector or to the Gal4-AD in pDEST22 vector using the Invitrogen Gateway^®^ Recombination Cloning Technology and following standard procedures. Once verified by sequencing, the final constructs were used to transform yeast following the LiAc/ssDNA/PEG protocol (Gietz 2014). Selection of transformed yeasts was performed by growing on Synthetic Defined (SD) medium without Leu (for yeast strain Y187 transformed with pDEST32 vectors) or Trp (for yeast strain Y2HGold transformed with pDEST22 vectors). Subsequently, diploid cells obtained by mating were selected on an SD medium lacking both Leu and Trp. Protein–protein interaction assays were performed in SD plates lacking Leu, Trp, and His in the presence of different concentrations of 3-amino triazole (Sigma-Aldrich).

### Promoter transactivation assay

To evaluate the activation by the candidate MYB and bHLH TFs of the two key genes for the epicatechin and catechin branches of the PA pathway, a genome fragment of 800–1200 bp upstream of the translation start codons of *ANR* and *LAR1* was PCR amplified from DNA samples of *L. corniculatus* "Charlii" using the primers reported in Table S2. These primers were designed on the *ANR* and *LAR1* promoters cloned by genome walking from the tetraploid S41 *L. corniculatus* genotype (Paolocci et al. unpublished results). The resulting PCR products were digested using *BamH*I and *Nco*I enzymes (Roche Diagnostics GmbH, Mannheim, Germany) and cloned into pGreenII 0800 LUC plasmid using T4 DNA Ligase (Promega). Both constructs, *ANR* promoter::Firefly luciferase reporter and *LAR* promoter::Firefly luciferase reporter were verified by sequencing and then transformed into *Agrobacterium tumefaciens* strain C58 containing the pSoup helper plasmid.

pDONR221 entry vectors containing *LcTT8* and the candidates MYBs *LcTT2b*, *LcMYBPA2*, and *LcMYB5* were recombined via LR reaction (LR Clonase II, Invitrogen) into pAlligator1 plasmid. Once verified by sequencing, the resulting cassettes were used to transform *A. tumefaciens* strain C58.

An aliquot of 0.5 OD (600 nm) from each *Agrobacterium* fresh culture was pellet by centrifugation (20 min at 5000 g) and resuspended in 2 ml of infiltration solution (10 mM MES, 10 mM MgCl2, and 100 µM acetosyringone). Combinations of *Agrobacterium* cultures for *Nicotiana benthamiana* infiltration were prepared by mixing equal amounts of each one. Young leaves of 2-week-old *N. benthamiana* plants (*n* = 3) cultivated in growth chambers were infiltrated. Leaf samples were extracted with Passive Lysis Buffer (Promega) two days post infiltration. Then the ratio of Firefly luciferase to *Renilla* luciferase fluorescence was measured using Dual-Glo® Luciferase Assay System (Promega) on a luminometer (Luminoskan Ascent, Thermo Scientific).

## Results

### Illumina Hiseq 1500 sequencing, de novo assembly of *L. corniculatus* and *L. tenuis* transcriptomes and functional annotation

After adapter sequences were trimmed and sequences shorter than 90 bases removed, 200,136,278 and 181,191,352 clean paired-end reads remained for de novo assembly by Trinity software. Reads assembly yielded 123,301 contigs (91,530 unigenes) with an average length of 880 bp from *L. corniculatus* and 109,953 contigs (80,911 unigenes) of 930 average bp length from *L. tenuis*. In *L. corniculatus,* 61,318 assemblies were > 500 bp and 35,369 > 1 kb, whereas in *L. tenuis*, 57,469 were > 500 bp and 34,021 > 1 kb. The quality of sequencing and assembly was verified by comparing the nucleotide sequences of *1αEF*, *PAL*, *CHS*, *DFR*, *ANS*, *ANR*, *LAR1*, *LAR2*, and *MATE1* genes from both *Lotus* spp. with those previously reported (Escaray et al. 2014, 2017); in all cases, the identity between these sequences was always higher than 95%.

Using BLASTx search in Uniprot databases, 94,005 (76.2%) and 82,760 (75.3%) transcripts were annotated from *L. corniculatus* and *L. tenuis*, respectively. Using a TransDecoder Software, 52,401 predicted proteins for *L. corniculatus* and 47,775 for *L. tenuis* were obtained, which were 43,854 (83.6%) and 39,688 (83.1%) after BLASTp search in Uniprot databases. Finally, predicted proteins were also functionally annotated by search in the pfam database; by this way, 34,191 (65.2%) and 31,275 (65.5%) sequences for *L. corniculatus* and *L. tenuis,* respectively, were annotated.

### Identification of putative PA regulators in *L. corniculatus* and *L. tenuis*

**Fig. 1:**
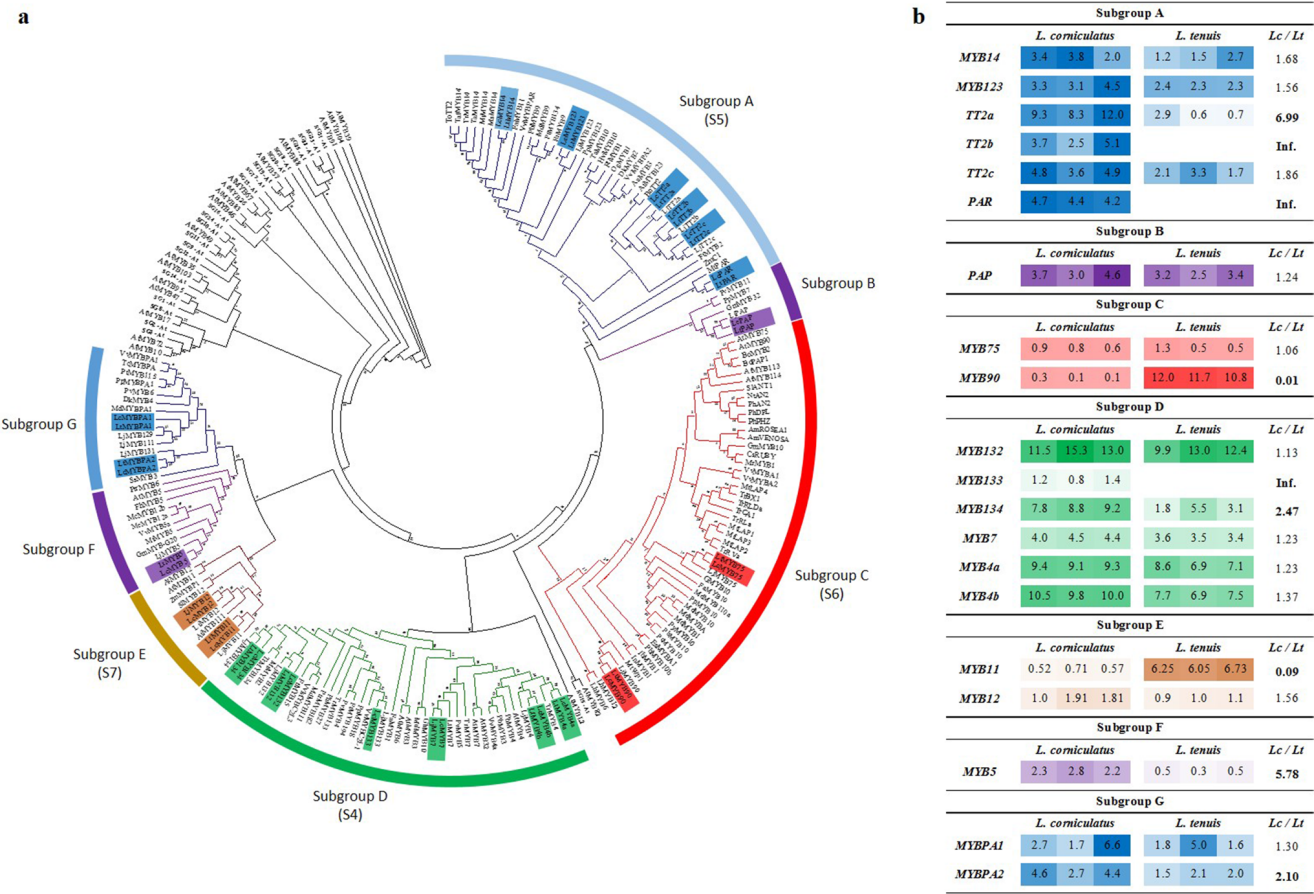
R2R3MYBs considered in the present study. **a** Evolutionary relationships of selected R2R3-MYB proteins. The Neighbor-Joining method inferred the evolutionary history (Saitou and Nei 1987). The optimal tree with the sum of branch length = 48.22591070 is shown. The evolutionary distances were computed using the p-distance method (Nei and Kumar 2000) and are in the units of the number of amino acid differences per site. The analysis involved 284 amino acid sequences. All positions with less than 95% site coverage were eliminated. There were a total of 162 positions in the final dataset. Evolutionary analyses were conducted in MEGA7 (Kumar et al. 2016). Reference sequences are detailed in Table S1. Branches in blue color indicate clusters that include MYB activators of proanthocyanidin (PA) biosynthesis; in purple MYBs reported to activate both PA and anthocyanin biosynthesis; in red MYB activators of anthocyanins; in light brown MYB activators of flavonoids; in green MYB repressors of both PA and anthocyanidins. In brackets are given the subgroups of the reference R2R3MYB Arabidopsis genes as designated in Strake et al. (2001). **b** FPKM (fragments per kilobase of exon model per million reads mapped) of selected MYBs from *L. corniculatus* and *L. tenuis* transcriptomes

A total of 280 and 257 putative MYB transcripts resulted from annotation and BLAST analyses of the *L. corniculatus* and *L. tenuis* transcriptomes, respectively. About 20 of them per species clustered with MYBs controlling PAs or anthocyanins in different plant species (Fig. S2). These were re-sequenced in both species and then employed to build a flavonoid-specific Neighbor-joining tree, which displayed seven subgroups, named from A to G (Fig. 1). MYB activators of PA biosynthesis formed subgroups A and G, the activators of PA, anthocyanins and flavonoids subgroups B and F, activators of anthocyanin subgroup C. In contrast, subgroup E included MYB11 and MYB12, reported to be as general activators of flavonoid biosynthetic genes, and subgroup D repressors of either PA and/or anthocyanin pathways.

Subgroup A, which corresponds to subgroup 5 (SG5) according to the MYB classification in Arabidopsis, included the *L. corniculatus* and *L. tenuis* homologs to LjTT2a, LjTT2b, LjTT2c, and TaMYB14, the contig LjSGA_029658 from *L. japonicus* reported as LjMYB123 (Shelton et al. 2012), and to MtPAR (Verdier et al. 2012). According to the FPKM values, the transcript levels of homologs to LjTT2a, LjTT2b, LjTT2c, and TaMYB14 were higher in *L. corniculatus* than in *L. tenuis* (Fig. 1b)*.* Coupled to the evidence that *TT2b* was not detected in the transcriptome of *L. tenuis*, the about seven-fold higher transcript levels of *TT2a* were also of interest. The level of *MYB123* transcripts was only slightly higher in the PA-rich *Lotus* spp., whereas no transcripts were found for PAR in the *L. tenuis* transcriptome. Subgroup G included MYBPA proteins with two candidates for each *Lotus* species here considered, named MYBPA1 and MYBPA2. Transcript levels of *MYBPA2* and *MYBPA1* were higher (2.1 fold) or slightly higher (1.3 fold) in *L. corniculatus* than in *L. tenuis*, respectively (Fig. 1b). The first MYBPA1 protein was characterized in grape as a PA regulator and belongs to a separate clade from the SG5 and SG6 R2R3 MYB genes (Bogs et al. 2007). Within subgroups B and F, there were the homologs to PAP from *L. japonicus* and MYB5 from various species, respectively. If the level of *PAP* transcripts was similar in the two transcriptomes, *MYB5* was 5.7 fold higher in *L. corniculatus* (Fig. 1b).

Subgroup C, corresponding to the SG6 in Arabidopsis, included MYB proteins related to anthocyanin regulation (Table S1), such as MYB75 and MYB90 (known as PAP1 and PAP2, Borevitz et al. 2000) with orthologues of both *MYB75* and *MYB90* found in both *Lotus* spp. transcriptomes. The FPKM values of *MYB75* were similar between the two species, and those of MYB90 were much higher in *L. tenuis* (80 fold).

Subgroup E included proteins, classified as SG7 in Arabidopsis, related to the regulation of flavonoid biosynthesis (Table S1) with two MYBs, MYB11 and MYB12 (Fig. 1a). *MYB11* was ten-fold more expressed in *L. tenuis*; on the contrary, the level of *MYB12* transcripts was slightly higher in *L. corniculatus* (Fig. 1b).

Since the present work was focused on identifying MYB activators of PAs in the foliage of *Lotus* spp, those belonging to subgroups A, B, C, F, and G were objects of further analyses. However, as shown in the phylogenetic tree, orthologues of repressors of PAs and/or anthocyanins were found in the two transcriptomes. More specifically, six *Lotus* MYBs here identified in subgroup D clustered with well-characterized repressors of the SG4 (Fig. 1a). This included MYBs highly similar to *T. repens* MYB132, MYB133, and MYB134, repressors of PAs and anthocyanins, and MYB7 and MYB4 repressors phenylpropanoid compounds (Albert 2015). In general, the transcript levels of these MYBs were slightly higher in *L. corniculatus* than in *L. tenuis* except for *MYB134*, which was 2.5-fold more expressed in *L. corniculatus* and of MYB133, which was not found in the *L. tenuis* transcriptome (Fig. 1b).

Regarding bHLH members, homologs from both *L. corniculatus* and *L. tenuis* were retrieved when their transcriptomes were scanned for TT8, GL3/EGL3, and TAN1 proteins. The levels of *TT8,* which showed 99% of identity with the LjTT8 and LcTT8 described previously (Escaray et al. 2017), were 5.2 fold higher in *L. corniculatus.* In contrast, those of *GL3/EGL3,* and *TAN1* were slightly higher in *L. tenuis* (Fig. S3). Finally, despite several reference sequences being used as queries, only a *TTG1* gene in both *Lotus* spp were retrieved. It showed high similarity with the *LcTTG1* gene previously cloned (Escaray et al. 2017) and *TTG1* from *L. japonicus*. The transcripts levels of *TTG1* in FPKM values were 13.2 ± 1.2 in *L. corniculatus* and 14.8 ± 0.8 in *L. tenuis* in the face of the fact that the former species showed 18.1 ± 2.2 mg PAs / g DM and the latter only 0.8 ± 0.2 mg.

### Relative expression of candidate genes for PA accumulation in different *Lotus* spp. accessions

The first three sample sets included “PA-rich” and “PA-poor” *Lotus* genotypes (Table S3). The expression of genes coding for structural enzymes of the PA pathway evaluated in the third sample set showed significantly higher levels for *CHS*, *DFR*, *ANS*, *ANR*, *LAR1*, *LAR2*, and *MATE* genes in “PA-rich” genotypes (Table S4). Better still, a positive correlation (*r* > 0.95; *P* value ≤  0.0001) emerged between the relative expression of all these genes and the levels of PAs. These findings keep and extend what emerged from the analyses of the first and second sample sets (Table 1, Escaray et al. 2014, 2017).

**Table 1:** Correlation between the relative expression levels of PA structural and regulatory genes and levels of PAs in PA polymorphic *Lotus* genotypes

		Correlation					
		1° cDNA set		2° cDNA set		3° cDNA set	
		*r*	*P*-value	*r*	*P*-value	*r*	*P*-value
Early genes	*PAL*	0.50	0.0300	-0.03	0.9200	0.50	0.0400
	*CHS*	*0.77*	*0.0002*	*0.59*	*0.0100*	*0.97*	< *0.0001*
Late genes	*DFR*	*0.99*	< *0.0001*	*0.77*	*0.0002*	*0.96*	< *0.0001*
	*ANS*	*0.97*	< *0.0001*	*0.72*	*0.0009*	*0.99*	< *0.0001*
	*ANR*	*0.96*	< *0.0001*	*0.68*	*0.0021*	*0.99*	< *0.0001*
	*LAR1*	*0.99*	< *0.0001*	*0.94*	< *0.0001*	*0.97*	< *0.0001*
	*LAR2*	*0.98*	< *0.0001*	*0.84*	< *0.0001*	*0.97*	< *0.0001*
	*TT12*	*0.99*	< *0.0001*	*0.90*	< *0.0001*	*0.98*	< *0.0001*
MYB	*TT2a*	*0.75*	*0.0003*	0.49	0.0400	*0.75*	*0.0006*
	*TT2b*	*0.96*	< *0.0001*	*0.93*	< *0.0001*	*0.61*	*0.0100*
	*TT2c*	0.55	0.0200	0.14	0.5800	*0.93*	< *0.0001*
	*MYB123*	*0.77*	*0.0002*	-0.04	0.8600	*0.75*	*0.0005*
	*MYB14*	*0.94*	< *0.0001*	0.58	0.1000	0.53	0.0300
	*PAR*	*0.96*	< *0.0001*	0.35	0.1500	-0.46	0.0600
	*PAP*	*0.74*	*0.0004*	0.13	0.6100	0.30	0.2500
	*MYBPA1*	*0.94*	< *0.0001*	0.62	0.0400	-0.06	0.8100
	*MYBPA2*	*0.97*	< *0.0001*	*0.92*	< *0.0001*	*0.95*	< *0.0001*
	*MYB5*	*0.94*	< *0.0001*	*0.93*	< *0.0001*	*0.95*	< *0.0001*
	*MYB75*	*0.95*	< *0.0001*	-0.19	0.4500	*0.61*	*0.0100*
	*MYB90*	0.54	0.0200	*0.75*	*0.0003*	0.44	0.0700
bHLH	*TT8*	*0.73*	*0.0013*	*0.86*	< *0.0001*	*0.97*	< *0.0001*
WDR	*TTG1*	-0.01	0.9700	-0.19	0.4500	0.48	0.0500

Samples within each cDNA set and relative gene expression levels are as reported in Table 1. PA levels expressed as mg of PA/g DW. Italic font indicates significant correlation (*P* value ≤ 0.01). Normal font indicates non significant correlation (*P* value ≤ 0.01)

**Fig. 2:**
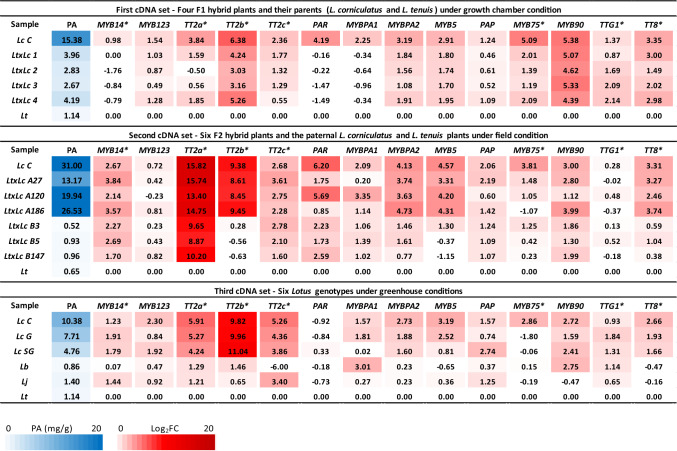
PA content and relative expression levels of regulatory genes in PA polymorphic *Lotus* spp. genotypes. The blue color indicates total proanthocyanidins (PA) levels. Red color indicate Log_2_ FoldChange of the gene expression levels, calculated using the 2^−(ΔΔCt)^ algorithm and refered to *L. tenuis* (mean from three biological replicates). Lc C: *L. corniculatus* “charlii”, LtxLc: *L. tenuis* x *L. corniculatus*, Lc G: *L. corniculatus* “Granada”, Lc SG: *L. corniculatus* “San Gabriel”, Lb: *L. burtii*, and Lj: *L. japonicus*

The same three sets of cDNA have been then employed to assess the expression levels of all candidate activators identified by the transcriptomic analysis reported above. The heatmap in Fig. 2 shows the relative expression of each of these genes and the levels of total PAs in each sample of the three cDNA sets. The statistics beyond this map are provided in Table S5. From the correlation analysis between PA and gene expression levels (Table 1), the only *MYB* of subgroup A that showed a significant correlation with PAs in any sample set was *TT2b*. *MYB14* and *PAR* showed a significant correlation with PAs only in the first set, *MYB123* and *TT2a* both in the first and third sets, whereas *TT2c* only in the third one.

Interestingly, the other *MYBs* that showed a significant correlation with PAs in any setting were *MYBPA2* (subgroup G; *r* ≥ 0.92, *P* value ≤ 0.0001) and *MYB5* (subgroup F; *r* ≥ 0.93, *P* value ≤ 0.0001). Focusing on genotypes that did not derive from interspecific hybridization, it turned out that the *MYBs* consistently upregulated in the three “PA-rich” *L. corniculatus* genotypes compared to the “PA-poor” *Lotus* ones were *TT2b* and *MYBPA2* only (Table S5). Regarding the bHLH and WDR partners, *TT8* always showed a significant correlation with PA in any comparison, whereas *TTG1* did not (Table 1).

The involvement of *TT2b*, *MYBPA2*, *MYB5*, and *TT8* in PA synthesis was confirmed by the positive correlation of their expression with those of *ANR*, *LAR1*, and *MATE* in any sample set analyzed (Table S6). Nevertheless, *MYB5* exhibited a positive correlation with *CHS* and *DFR, MYBPA2* with *DFR,* and *TT8* with *ANS* in these sets. A positive correlation also emerged between the expression of *TT2a* and that of *ANS*, *ANR*, and *LAR1* (*P* value ≤ 0.01) in any sample set*.*

### PA and gene expression levels in *L. corniculatus* seedlings

The levels of PAs in *L. corniculatus* plants depend on tissues and organs (Escaray et al. 2014). In the seedlings of *L. corniculatus* “Charlii”, PA-accumulating cells were present through mesophyll and around the vascular tissues since their first leaves. Still, they were absent in the cotyledons (Fig. S4). Thus, the relative expression of structural genes related to PAs, *TTG1*, *TT8*, and different *MYBs* was compared between the seedlings shoot apex and the cotyledons (Fig. S4b, c). All genes coding for late structural enzymes of the PA pathway showed higher relative expression in shoot apex than in cotyledon, particularly *LAR1* and *LAR2,* 92.6 and 192.8 folds, respectively. Concerning the candidates of MBW complex, no difference was observed for *TTG1,* whereas *TT8* was 46.9 folds higher expressed in the shoot apex. Likewise, *MYB14*, *MYB123, TT2a*, *TT2b*, *TT2c*, *PAR*, *PAP*, *MYBPA2*, and *MYB5* were more expressed in shoot apex than in cotyledons, with fold changes ranging from 287.0 (*TT2a*) down to 2.99 (*MYB5*). No differences were observed for both *MYB*s of subgroup C (*MYB75* and *MYB90*), whereas the relative expression of *MYBPA1* was higher in cotyledons.

### Functional evaluation of the interaction between selected *Lotus* MYBs and bHLHs by Y2H assay

**Fig. 3:**
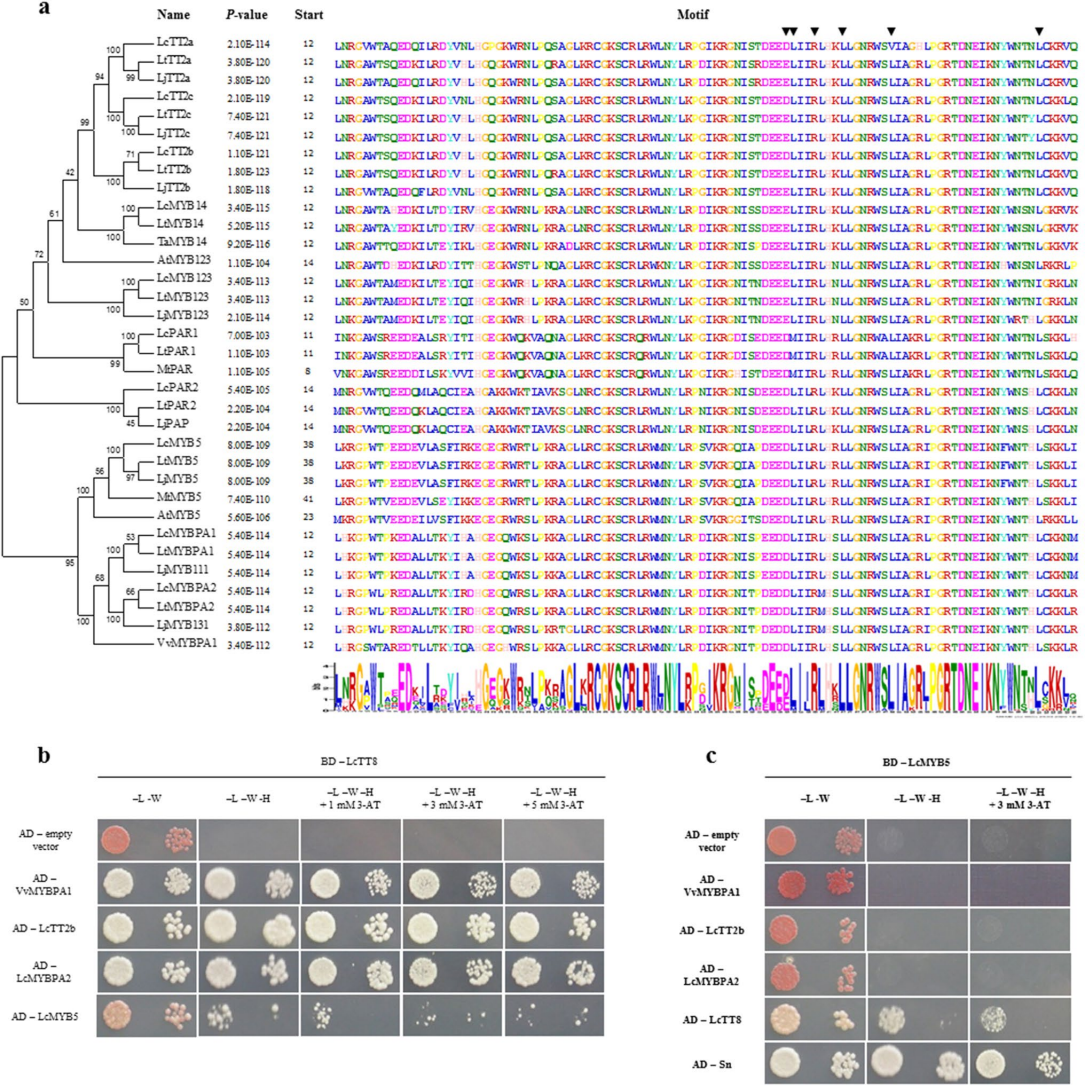
MYB-bHLH interactions. **a** Phylogenetic tree of selected MYBs and R2R3 conserved motif (MEME). Black triangles indicate the [*D*/*E*]*L*_x2_[*R*/*K*]_x3_*L*_x6_*L*_x3_*R* motifs important for the interaction with bHLH proteins. **b** Interactions between LcTT8 fused to GAL4 DNA binding domain (BD) and VvMYBPA1, LcTT2b, LcMYBPA2, or LcMYB5 fused to GAL4 activation domain (AD) evaluated by yeast two-hybrid assay. Two serial dilutions per yeast clone grown in control media (-L -W), selective media (-L -W -H) and selective media supplemented with 3-aminotriazole (3-AT) at three different concentrations (1, 3 and 5 mM) are shown. **c** Interactions of LcMYB5 fused to the GAL4 BD and LcTT2b, LcMYBPA2, VvMYBPA1, LcTT8, or Sn fused to the GAL4 AD evaluated by yeast two-hybrid assay. MYBPA1 from *V. vinifera* and Sn from *Z. mays* were used as MYB and bHLH positive control, respectively. Dilutions are as in **b**

MYBs have to interact with bHLH proteins to form an active MBW complex. All the R2R3MYBs are likely involved in the regulation of flavonoids retrieved from the transcriptomes of the two *Lotus* spp. showed the conserved amino acid signature ([*D*/*E*]*L*_x2_[*R*/*K*]_x3_*L*_x6_*L*_x3_*R*) for the interaction with bHLH proteins (Fig. 3a). The yeast two-hybrid assays were thus employed to experimentally confirm the interaction between *L. corniculatus* TT8 and the *L. corniculatus* MYBs that showed a significant correlation with the PA levels in any sample set investigated TT2b, MYBPA2, and MYB5. As a control, MYBPA1 from grapes and Sn from maize were used. The first is an important regulator of PA biosynthesis in grapes which induced a metabolic diversion from anthocyanins to PAs in transgenic tobacco flowers (Bogs et al. 2007; Passeri et al. 2017); the second is an activator of anthocyanins in maize, which promoted the expression of the PA genes, thereby increasing the number of PA accumulating leaf cells and the overall PA levels, when ectopically expressed in *L. corniculatus* (Damiani et al. 1999; Paolocci et al. 2007). All the LcMYBs, but one (LcMYB5), strongly interacted with bHLH members (LcTT8) as much as VvMYBPA1 did. Interestingly, LcMYB5 weakly interacted with LcTT8, but it did it strongly with Sn (Fig. 3b, c). In *M. truncatula* MYB5 forms with MYB14, TT8, and WD40-1, a quaternary MBW complex to activate the *ANR* and *LAR* promoters (Liu et al. 2014), thus the Y2H assays were performed to test if the presence of other MYBs could mediate the interaction of MYB5 with TT8. This hypothesis had to be ruled out, at least for what concerns LcTT2b, LcMYBPA2, and VvMYBPA1, since LcMYB5 did not interact with any of these major PA regulators (Fig. 3c).

### Functional analysis of the transactivation of the *ANR* and *LAR1* promoters by candidate LcMYB and LcbHLH PA regulators

To test whether and to what extent the candidate MYBs and bHLH regulate the transcription of the critical genes for PA accumulation in *Lotus* spp., the promoters of the genes coding for functional enzymes in catalyzing the synthesis of catechin and epicatechin units were cloned from both *L. corniculatus* and *L. tenuis* species. These genes were *ANR* and *LAR1* but not *LAR2,* since the enzyme coded by this gene could not yield catechins from leucoanthocyanidins (Paolocci et al. 2007). The about 430 bp long regions upstream of the coding sequence of *ANR* of *L. tenuis* and *L. corniculatus* showed a high level of identity between each other (98.6%). PLACE predicted two bHLH-recognizing elements (BREs) and one MYB-recognizing element (MRE) in the *ANR* promoter from both species, respectively (Fig. S5). The about 710 bp long regions upstream of the coding sequence of *L. tenuis* and *L. corniculatus LAR1* also showed high identity (93.9%). Additionally, the *LAR1* promoter of the two species showed four and two conserved BREs and MREs *cis*-elements, respectively. Notably, the two MREs overlapped with the two central BREs. It is worth noting that all the BREs and MREs found in the promoters of both *ANR* and *LAR1* genes from *L. corniculatus* and *L. tenuis* were also found in the promoters of the same genes from *L. japonicus* (Fig. S5)*.* This evidence paves the way for experiments to test the hypothesis suggesting that different transcriptional rates of these two genes between PA-rich and poor *Lotus* spp. does not depend on mutations of their regulatory sequences.

**Fig. 4:**
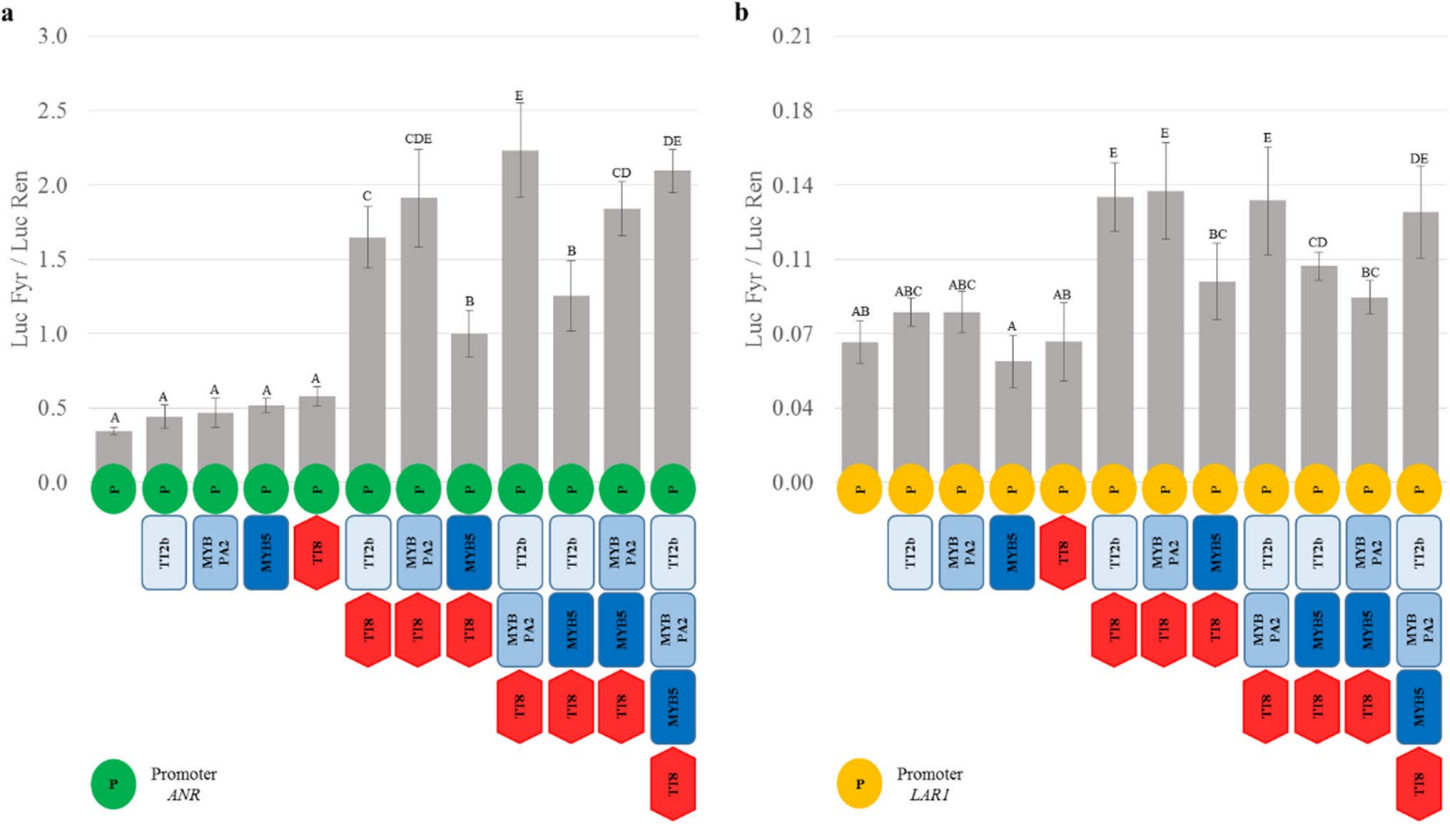
Promoter transactivation assays. **a** Activation of *ANR* promoter by the *L. corniculatus* MYBs. **b** activation of *LAR1* promoters. MYBs are given in different blue color scales, TT8 in red. Means were obtained from three biological replicates; different letters among bars indicate significant differences (*P* value ≤ 0.01; Duncan's test)

*N. benthamiana* leaves were employed to test the capacity of the selected regulators to transactivate the promoters of *ANR* and *LAR1* from the diploid *L. corniculatus* plant (Fig. 4). The infiltration of a single MYB, whatever it was, or of TT8 alone was not sufficient to significantly activate the luciferase reporter gene when driven either by *ANR* or *LAR1* promoter. Conversely, this activation was achieved when TT8 was co-infiltrated with an MYB, regardless of whether the MYB being tested was TT2b, MYBPA2, or MYB5. Yet, the activation of both *ANR* and *LAR1* promoters was significantly higher in leaves co-infiltrated with TT2b-TT8 or MYBPA2-TT8 than with MYB5-TT8, and the luciferase signal was always about an order of magnitude lower when the reporter gene was driven by the promoter of *LAR1* than *ANR,* regardless of the combination of TFs used.

To test whether multiple MYBs cooperate to activate these promoters, the combinations of two or three MYBs with TT8 were evaluated. The activation of *ANR* increased when TT2b and MYBPA2 were simultaneously employed with respect to the sole TT2b; the same did not occur for *LAR1* (Fig. 4). Conversely, when MYB5 replaced one of these two MYBs, the luciferase signals decreased; regardless of the promoter used. This decrement was more pronounced when MYB5 was tested with TT2b than with MYBPA2 on *ANR* promoters. Adding MYBPA2 to TT2b-MYB5-TT8 raised the luciferase signal to values found with MYPA2-TT8 and MYBPA2-TT2b-TT8 combinations. Conversely, on the *LAR1* promoter, the decrement due to the presence of MYB5 was slightly more severe when it was used in combination with MYBPA2 and TT8 than with TT2b and TT8, and the negative effect of MYB5 was rescued when it was co-infiltrated with MYBPA2-TT2b and TT8 (Fig. 4).

## Discussion

### The expression of *TT2b*, *MYBPA2*, *MYB5*, and *TT8* correlates with the levels of PA in *Lotus* spp.

The synthesis of PAs is mainly regulated at the transcriptional level by the ternary MBW complex (Baudry et al. 2004). TT2 from Arabidopsis is the best-studied MYB controlling PA biosynthesis, which in this species occurs only in the seed coat and via the ANR branch (Nesi et al. 2001; Abrahams et al. 2003). Three *TT2* orthologs (*TT2a*, *TT2b*, and *TT2c*) have been characterized in *L. japonicus* (Yoshida et al. 2010b) and *L. corniculatus* (Escaray et al. 2017). The three LjTT2s are involved in the induction of *LjANR* but not of *LjLAR*, and they show different expression patterns and interaction abilities with TT8 and TTG1 (Yoshida et al. 2008, 2010b). Additionally, complementation analysis of Arabidopsis *tt2* mutants showed that *M. truncatula* PAR and MYB14 proteins are the functional orthologs of TT2 and that AtMYB5 but not MtMYB5 rescues PAs in the seeds of this mutant (Liu et al. 2014; Xu et al. 2014). MYB14 has also been characterized in *Trifolium* spp (Hancock et al. 2012) and *L. corniculatus,* but its expression did not mirror the levels of PA in the *Lotus* genotypes tested (Escaray et al. 2017). To search for additional PA players and herbage-specific MBW complexes, here we compared the shoot transcriptomes of two *Lotus* species, *L. corniculatus* and *L. tenuis,* which displayed a marked difference in PA accumulation in these organs. The commitment of candidate genes in this trait has been confirmed by studying their expression levels in *L. corniculatus* x *L. tenuis* hybrids and their progeny and *Lotus* species and organs with different commitments for PA synthesis grown under different environmental conditions. Our phylogenetic analysis has sorted about 20 MYBs per transcriptome into the seven clusters (named from A to G) related to PA or anthocyanin regulators. Within subgroup A, only TT2b shows a positive correlation with *ANR*, *LAR1*, and *MATE* expression and with the levels of PAs in any sample set and condition investigated. Putative *Lotus* PA regulators are also found in clusters F and G, containing the VvMYBPA and AtMYB5 reference proteins, respectively. The *Lotus* MYB5 positively correlates with the levels of PA accumulation and the expression of genes from *CHS* down to *MATE* in any set investigated. MYBPAs from different species are known as solid activators of the PA pathway (Bogs et al. 2007; Akagi et al. 2009; Ravaglia et al. 2013). MYBPA1 from grape rescues PA accumulation in Arabidopsis *tt2* mutant. However, Arabidopsis does not have a MYBPA1 orthologue, and it activates the promoters of two PA-specific biosynthetic genes, VvLAR and VvANR. Still, it could not turn on *VvUFGT*, which is necessary for anthocyanin biosynthesis (Bogs et al. 2007). Additionally, the *Lotus* MYBPA1 and MYBPA2 cluster with MYBPA1.1 from *Vaccinium myrtillus* (Fig. S6) which has been very recently shown to exert a dual role in co-regulating PA biosynthesis and anthocyanin biosynthesis (Lafferty et al. 2022). More recently, Jin et al. (2022) reported a new MYB, OvMYBPA2, whose expression correlates with PA accumulation in sainfoin (*Onobrychis viciifolia)*; however, this MYB clusters in subgroup A together with TT2s and MYB14, thus *Lotus* MYBPAs and the sainfoin OvMYBPA2 are not orthologs (Fig. S6). Therefore, to the best of our knowledge, this is the first report describing the presence of MYBPA orthologs in legumes. Of the two *MYBPA* genes present, only *MYBPA2* shows a strong correlation with the levels of PAs in any sample set investigated. Its expression positively correlates with that from *DFR* to downstream genes.

The transcriptomic data also suggest that PA accumulation in *Lotus* occurs independently from the activation of MYBs that promote anthocyanins in other genera since none of the candidate MYBs, MYB75, MYB90, or PAP1 and PAP2 (named after those of *L. japonicus* (Yoshida et al. 2010a) are upregulated in *L. corniculatus*. Moreover, in keeping with the negligible, if any, accumulation of anthocyanins in the two species under investigation, transcripts relative to the anthocyanin activators found in other legumes*,* such as LAP1-4 in *M. truncatula* and Tr-RED LEAF, Tr-RED V, Tr-CA1, Tr-RED LEAF DIFFUSE and Tr-BX1 proteins in *T. repens* (Peel et al. 2009; Albert et al. 2014), were not found. TT8 is likely the only bHLH member that is pivotal in controlling PAs in *Lotus* spp., as it is the sole DE *bHLH* gene between the two initial transcriptomes. Its expression correlates with *ANS*, *ANR*, *LAR1*, *LAR2* and *MATE* genes, with PAs' total levels in any sample set investigated. The present study also shows that, differently from other legume species, *TTG1* expression correlates neither with PAs nor with the expression of the structural genes and any of the MYB and bHLH regulators. In *M. truncatula* it has been reported that *MtWD40-1,* which complements the Arabidopsis *ttgg1* mutant, is mainly expressed in the seed coat as well as *MtPAR*, which, in turn, is sufficient to activate *MtWD40-1* transcription in one hybrid yeast assay (Pang et al. 2009; Verdier et al. 2012). The combinations of MtPAR, MtLAP1, MtTT8, and MtWD40-1 also activate the promoter of *MtTT8* (Li et al. 2016). The lack of correlation between the expression of *TTG1* or *PAR* with that of *TT8* and with the overall levels of PAs coupled with the evidence that *LAP*s are not among the genes present in our transcriptomes suggests that in the herbage of forage legumes, the presence of *TTG1* is dispensable for the correct assemblage of the PA- specific protein complexes. Alternatively, the basal levels of *TTG1* might be sufficient to ensure such complexes' formation. Likewise, MBW complexes operating in seeds differ from those operating in vegetative organs. This could partially explain why the ectopic expression of PA activators from *M. truncatula* and Arabidopsis was insufficient to produce bloat-safe forage legumes.

### MYB5 interferes with TT2b-TT8 and MYBPA2-TT8 mediated activation of the *ANR* and *LAR* promoters

Xu and collaborators (2014) have remarked that four different MBW complexes in Arabidopsis (TT2-TT8-TTG1, MYB5-TT8-TTG1, TT2-EGL3-TTG1, and TT2-GL3-TTG1) are involved, in a tissue-specific manner, in the transcriptional regulation of LGB genes related to PA biosynthesis. Likewise, the present study reveals that different MBW complexes, with likely partially overlapping functions, might be involved in this regulation in *Lotus* spp. Several could be the MYB partners of these complexes, namely all those present in subgroup A and MYBPA and MYB5 from groups G and F, respectively. We cannot rule out the formation of even quaternary complexes in which activators and repressors are involved, the last providing feedback regulation to MBW complexes (Albert 2015). Notwithstanding, our data suggest that the complexes in which are present TT2b, MYBPA2 for the MYB component, and TT8 for the bHLH are the ones that more strongly promote PA biosynthesis in *Lotus* herbage. Additionally, our assays unveil a different commitment among MYBs to interact with TT8. The findings that TT2b and MYBPA2 strongly interact in vitro with TT8 without TTG1 reinforces our contention that TTG1 is either dispensable or its basal levels sufficient to ensure the correct assemblage of the MYBPA2-TT8 and TT2b-TT8 PA complexes. However, this does not hold for MYB5. This protein interacts weakly with TT8 but firmly with Sn. Since in most of the plants studied, the WDR proteins interact with the bHLH TFs only (Grotewold et al. 2000; Dubos et al. 2008; An et al. 2012), and the maize bHLHs do not require the orthologs of TTG1 to form complexes with MYBs, we infer that MYB5 can bind bHLH and promote the transcription of PA specific genes only in an environment where TTG1 is expressed. From the transactivation assays in *N. benthamiana* leaves, we can also argue that *ANR* and *LAR1* promoters are activated when either TT2b-TT8 or MYBPA2-TT8 proteins are co-expressed. Strikingly, when transfected with TT8, MYB5 activates, although to a far less extent than the other two MYBs, the promoter of *ANR* but not that of *LAR1*.

Conversely, by transfecting Arabidopsis protoplasts, Liu and colleagues (2014) have shown that MtMYB5 alone is sufficient to transactivate both *MtANR* and *MtLAR* promoters and that the addition of TT8 can enhance this effect but only on *ANR* promoter. The different outcomes from these studies can stem from the various regulatory elements in the promoters of the two species and/or the different host systems employed. The presence of the endogenous MBW partners could mediate these MYB-bHLH interactions. In this context, the endogenous WDR40 partner expressed in *N. benthamiana* leaves (Albert et al. 2014; Montefiori et al. 2015) might be responsible for the functional assemblage MYB5-TT8 complex and the following activation of the *LcANR* promoter.

The finding that MYBPA2 induces the transactivation of both *ANR* and *LAR1* promoters provides functional evidence that this newly identified MYB plays a crucial role in activating both PA branches in *Lotus* spp. Still better, MYBPA2 amplifies the effects of TT2b on *ANR* promoter. Notwithstanding, MYB5 compromises the transactivation of activation of *ANR* and *LAR1* by MYBPA2 and TT2b. This outcome is somewhat unexpected since MtMYB5 can synergistically act with another MYB activator (i.e., MtMYB14) to promote *ANR* and *LAR* transcription (Liu et al. 2014).

### Bottleneck and perspectives for engineering PAs in forage legumes

The approach and the experimental material employed have allowed us to: (a) add new MYB players in the regulation of PA pathway in forage legumes; (b) refine our previous contention, stemming from transgenic approaches, that *ANR* and *LAR1* genes are tightly co-regulated (Paolocci et al. 2007, 2011) and (c) highlight striking differences concerning the regulation of this pathway in *Lotus* versus other genera of forage legumes. Here, we provide compelling data showing that another player, MYBPA2, adds to the MYBs known to control PA biosynthesis in legumes. Better still, MYBPA2 seems to play a more relevant role than TT2b on the activation of *ANR* promoter, which, in turn, is more responsive to the transfection with MYB5, MYBPA2, and/or TT2b along with TT8 proteins than *LAR1* promoter. It also appears peculiar the role of MYB5: it likely plays a role as a general activator of the flavonoid pathways. It only promotes *ANR* transcription in *Lotus* organs/species when either TT2b or MYBPA2 are absent. Conversely, it acts as a passive repressor, likely because it recruits other components on the cis-elements of PA genes when either MYBPA2 or TT2 are present. However, such an effect seems titration-dependent because it is reverted when MYBPA2 and TT2b are expressed. The putative dampening effect of an MYB activator could represent an additional means by which plant cells and organs control the biosynthesis of these pigments. In turn, because of the dampening effect, the pyramiding of multiple TFs might not always be adequate to engineer the biosynthesis of PAs.

## Conclusions

The plethora of activator and repressor MYBs found in the two *Lotus* transcriptomes calls for many MBW complexes underlying the biosynthesis of PAs in forage legumes. These complexes might also differ in composition according to specific developmental windows and growing conditions. This evidence aligns with the complexity of protein interactions, regulatory loop, and gene hierarchy underlying the biosynthesis of PAs described in other genera (Lafferty et al. 2022). Nevertheless, by comparing multiple PA-polymorphic *Lotus* genotypes grown under different environmental conditions, we show that TT8 for bHLHs and MYBPA2, along with TT2b for MYBs, are the significant determinants of PA biosynthesis in the herbage of *Lotus* spp. The ectopic expression under different combinations of these TFs, driven by leaf-specific or constitutive promoters to bypass the problem of any hierarchical regulation of these genes, is ongoing in *L. tenuis* and alfalfa. The goal is to verify whether and to what extent the co-expression of potent regulators of the *ANR* and *LAR* branches of the PA pathway will be sufficient to build PAs in species depleted or not naturally committed to synthesizing these compounds in the herbage.

### Supplementary Information

Below is the link to the electronic supplementary material.

Supplementary file1 (DOCX 57 KB)

Supplementary file2 (PPTX 471 KB)

Supplementary file3 (DOCX 321 KB)

Supplementary file4 (DOCX 166 KB)

Supplementary file5 (DOCX 609 KB)

Supplementary file6 (DOCX 110 KB)

Supplementary file7 (DOCX 17 KB)

Supplementary file8 (DOCX 49 KB)

Supplementary file9 (DOCX 144 KB)

Supplementary file10 (DOCX 18 KB)

Supplementary file11 (DOCX 19 KB)

Supplementary file12 (DOCX 38 KB)

## Data Availability

The RNA-seq data that support the findings of this study are available in NCBI BioProject, reference number [PRJNA609966], BioSample accessions SAMN14267684 (*L. corniculatus*) and SAMN14267774 (*L. tenuis*). The sequences of *L.corniculatus* and *L.tenuis ANR* and *LAR1* promoters are deposited in GenBank under the accession numbers OM362813-OM362816.

## Abbreviations

ANR: Anthocyanins reductase
bHLH: Basic helix-loop-helix
BRE: BHLH-recognizing elements
LAR: Leucoanthocyanidin reductase
MBW: MYB-bHLH-WDR complex
MRE: MYB- recognizing elements
Pas: Proanthocyanidins
TFs: Transcription factors
Y2H: Yeast two-hybrid assay
WDR: WD-repeat
