# Supplementary material for: Multiple bHLH/MYB-based protein complexes regulate proanthocyanidin biosynthesis in the herbage of Lotus spp
Source: Planta. 2023 Dec 2;259(1):10. doi: 10.1007/s00425-023-04281-2 (PMC10693531; doi:10.1007/s00425-023-04281-2)
Supplement: Supplementary file 1 — Supplementary file1 (DOCX 57 KB) [file 425_2023_4281_MOESM1_ESM.docx]

**Supplemental Table 6.** Correlation between the relative expression levels of *MYB*, *bHLH* and *WDR* genes and those of genes coding for key structural enzymes of the PA pathway in PA polymorphic *Lotus* genotypes. Samples within each cDNA set and relative gene expression levels are as as reported in Table 1. Pearson correlation coefficient (*r*) were obtained from three biological replicates; dark grey blocks indicate significant correlations (*P*-value ≤ 0.01).
