# Supplementary material for: Multiple bHLH/MYB-based protein complexes regulate proanthocyanidin biosynthesis in the herbage of Lotus spp
Source: Planta. 2023 Dec 2;259(1):10. doi: 10.1007/s00425-023-04281-2 (PMC10693531; doi:10.1007/s00425-023-04281-2)
Supplement: Supplementary file 2 — Supplementary file2 (PPTX 471 KB) [file 425_2023_4281_MOESM2_ESM.pptx]

## Slide 1
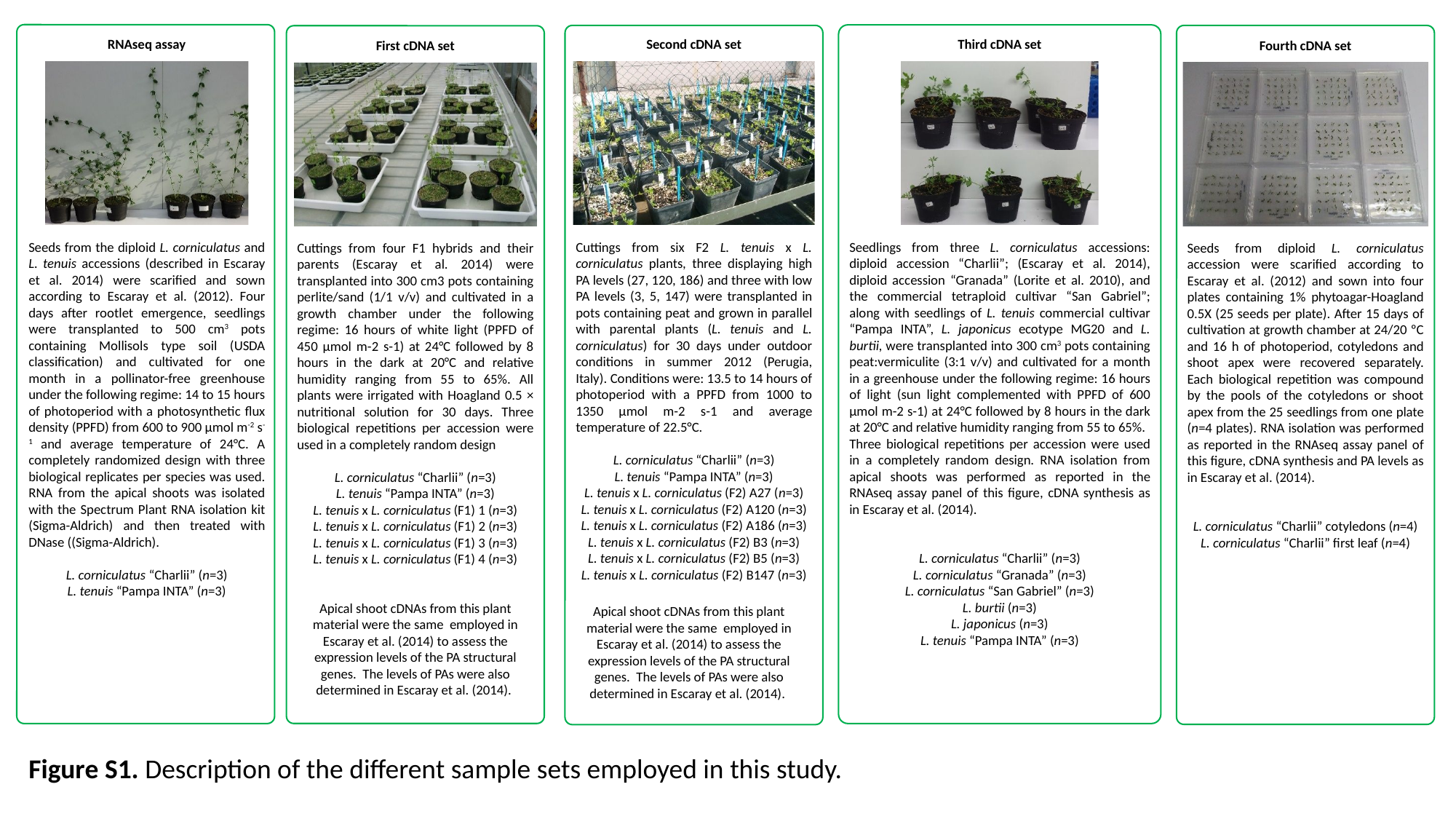

RNAseq assay
Seeds from the diploid L. corniculatus and L. tenuis accessions (described in Escaray et al. 2014) were scarified and sown according to Escaray et al. (2012). Four days after rootlet emergence, seedlings were transplanted to 500 cm3 pots containing Mollisols type soil (USDA classification) and cultivated for one month in a pollinator-free greenhouse under the following regime: 14 to 15 hours of photoperiod with a photosynthetic flux density (PPFD) from 600 to 900 μmol m-2 s-1 and average temperature of 24°C. A completely randomized design with three biological replicates per species was used. RNA from the apical shoots was isolated with the Spectrum Plant RNA isolation kit (Sigma-Aldrich) and then treated with DNase ((Sigma-Aldrich).
L. corniculatus “Charlii” (n=3)
L. tenuis “Pampa INTA” (n=3)
Third cDNA set
Seedlings from three L. corniculatus accessions: diploid accession “Charlii”; (Escaray et al. 2014), diploid accession “Granada” (Lorite et al. 2010), and the commercial tetraploid cultivar “San Gabriel”; along with seedlings of L. tenuis commercial cultivar “Pampa INTA”, L. japonicus ecotype MG20 and L. burtii, were transplanted into 300 cm3 pots containing peat:vermiculite (3:1 v/v) and cultivated for a month in a greenhouse under the following regime: 16 hours of light (sun light complemented with PPFD of 600 μmol m-2 s-1) at 24°C followed by 8 hours in the dark at 20°C and relative humidity ranging from 55 to 65%.
Three biological repetitions per accession were used in a completely random design. RNA isolation from apical shoots was performed as reported in the RNAseq assay panel of this figure, cDNA synthesis as in Escaray et al. (2014).
L. corniculatus “Charlii” (n=3)
L. corniculatus “Granada” (n=3)
L. corniculatus “San Gabriel” (n=3)
L. burtii (n=3)
L. japonicus (n=3)
L. tenuis “Pampa INTA” (n=3)
Second cDNA set
Cuttings from six F2 L. tenuis x L. corniculatus plants, three displaying high PA levels (27, 120, 186) and three with low PA levels (3, 5, 147) were transplanted in pots containing peat and grown in parallel with parental plants (L. tenuis and L. corniculatus) for 30 days under outdoor conditions in summer 2012 (Perugia, Italy). Conditions were: 13.5 to 14 hours of photoperiod with a PPFD from 1000 to 1350 μmol m-2 s-1 and average temperature of 22.5°C.
L. corniculatus “Charlii” (n=3)
L. tenuis “Pampa INTA” (n=3)
L. tenuis x L. corniculatus (F2) A27 (n=3)
L. tenuis x L. corniculatus (F2) A120 (n=3)
L. tenuis x L. corniculatus (F2) A186 (n=3)
L. tenuis x L. corniculatus (F2) B3 (n=3)
L. tenuis x L. corniculatus (F2) B5 (n=3)
L. tenuis x L. corniculatus (F2) B147 (n=3)
Fourth cDNA set
Seeds from diploid L. corniculatus accession were scarified according to Escaray et al. (2012) and sown into four plates containing 1% phytoagar-Hoagland 0.5X (25 seeds per plate). After 15 days of cultivation at growth chamber at 24/20 ºC and 16 h of photoperiod, cotyledons and shoot apex were recovered separately. Each biological repetition was compound by the pools of the cotyledons or shoot apex from the 25 seedlings from one plate (n=4 plates). RNA isolation was performed as reported in the RNAseq assay panel of this figure, cDNA synthesis and PA levels as in Escaray et al. (2014).
L. corniculatus “Charlii” cotyledons (n=4)
L. corniculatus “Charlii” first leaf (n=4)
First cDNA set
Cuttings from four F1 hybrids and their parents (Escaray et al. 2014) were transplanted into 300 cm3 pots containing perlite/sand (1/1 v/v) and cultivated in a growth chamber under the following regime: 16 hours of white light (PPFD of 450 μmol m-2 s-1) at 24°C followed by 8 hours in the dark at 20°C and relative humidity ranging from 55 to 65%. All plants were irrigated with Hoagland 0.5 × nutritional solution for 30 days. Three biological repetitions per accession were used in a completely random design
L. corniculatus “Charlii” (n=3)
L. tenuis “Pampa INTA” (n=3)
L. tenuis x L. corniculatus (F1) 1 (n=3)
L. tenuis x L. corniculatus (F1) 2 (n=3)
L. tenuis x L. corniculatus (F1) 3 (n=3)
L. tenuis x L. corniculatus (F1) 4 (n=3)
Apical shoot cDNAs from this plant material were the same employed in Escaray et al. (2014) to assess the expression levels of the PA structural genes. The levels of PAs were also determined in Escaray et al. (2014).
Apical shoot cDNAs from this plant material were the same employed in Escaray et al. (2014) to assess the expression levels of the PA structural genes. The levels of PAs were also determined in Escaray et al. (2014).
Figure S1. Description of the different sample sets employed in this study.
