# Supplementary material for: Multiple bHLH/MYB-based protein complexes regulate proanthocyanidin biosynthesis in the herbage of Lotus spp
Source: Planta. 2023 Dec 2;259(1):10. doi: 10.1007/s00425-023-04281-2 (PMC10693531; doi:10.1007/s00425-023-04281-2)
Supplement: Supplementary file 3 — Supplementary file3 (DOCX 321 KB) [file 425_2023_4281_MOESM3_ESM.docx]

**
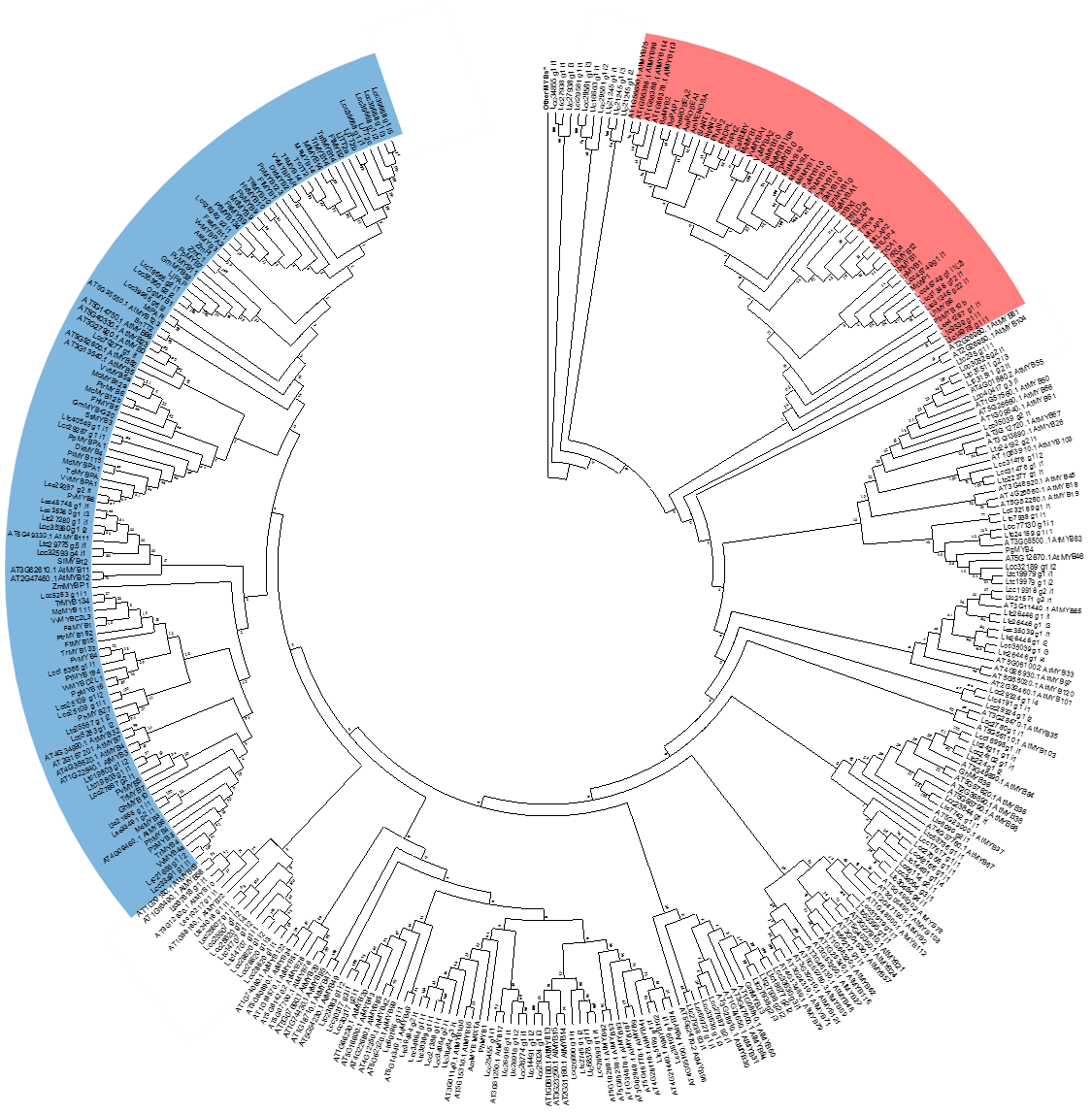
**

**Supplemental Figure 2.** Evolutionary relationships of MYB proteins. Phylogenetic tree of the MYB proteins listed in Supplemental Table S1 along with those retrieved from the two *Lotus* transcriptomes. The evolutionary history was inferred using the Neighbor-Joining method (Saitou and Nei, 1987). The optimal tree with the sum of branch length = 292.77984339 is shown. The evolutionary distances were computed using the p-distance method (Nei and Kumar, 2000) and are in the units of the number of amino acid differences per site. The analysis involved 1114 amino acid sequences. All ambiguous positions were removed for each sequence pair. There were a total of 3603 positions in the final dataset. Evolutionary analyses were conducted in MEGA7 (Kumar et al. 2016). In red are highlighed MYB proteins related to anthocyanin regulation; in blue those related to proanthocyanidin regulation.
