# Supplementary material for: Multiple bHLH/MYB-based protein complexes regulate proanthocyanidin biosynthesis in the herbage of Lotus spp
Source: Planta. 2023 Dec 2;259(1):10. doi: 10.1007/s00425-023-04281-2 (PMC10693531; doi:10.1007/s00425-023-04281-2)
Supplement: Supplementary file 4 — Supplementary file4 (DOCX 166 KB) [file 425_2023_4281_MOESM4_ESM.docx]

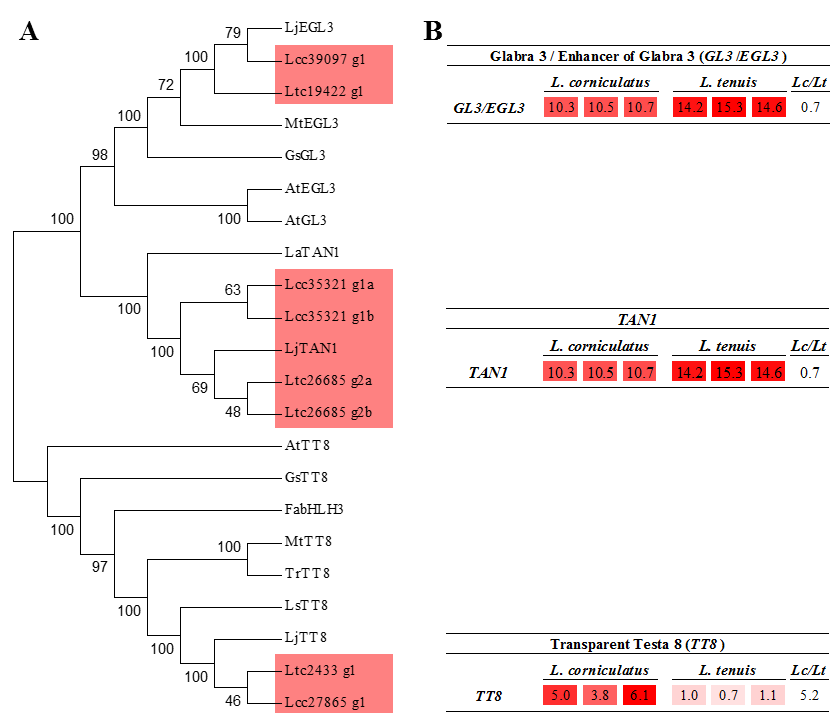


**a**

**b**

**Supplemental Figure 3.** *Lotus* bHLH proteins considered in this study**. a** Evolutionary relationships of bHLH proteins. The evolutionary history was inferred using the Neighbor-Joining method (Saitou and Nei, 1987). The optimal tree with the sum of branch length = 2.52032777 is shown. The evolutionary distances were computed using the p-distance method (Nei and Kumar, 2000) and are in the units of the number of amino acid differences per site. The analysis involved 294 amino acid sequences. All ambiguous positions were removed for each sequence pair. There were a total of 1012 positions in the final dataset. Evolutionary analyses were conducted in MEGA7 (Kumar *et al.*, 2016). Reference sequences are detailed in Supplemental Table 1. Branches in blue colour indicate groups that include MYB sequences that has been described as positive regulators of genes related to proanthocyanidin (PA) biosynthesis; in purple colour are the groups that include both MYBs, those than acts as PA-gene related activators and those that has been related with anthocyanin biosinthesis regulation; red indicate MYBs that activate anthocyanin-related genes; light brown indicate MYBs that regulate positively the flavonoid related genes; and green branches indicate MYBs that has been described as repressor or both PA and anthocyanidin related genes. **b** FPKM (fragments per kilobase of exon model per million reads mapped) of selected MYBs from *Lotus corniculatus* and *L. tenuis* transcriptome analysis.
