# Supplementary material for: Multiple bHLH/MYB-based protein complexes regulate proanthocyanidin biosynthesis in the herbage of Lotus spp
Source: Planta. 2023 Dec 2;259(1):10. doi: 10.1007/s00425-023-04281-2 (PMC10693531; doi:10.1007/s00425-023-04281-2)
Supplement: Supplementary file 5 — Supplementary file5 (DOCX 609 KB) [file 425_2023_4281_MOESM5_ESM.docx]

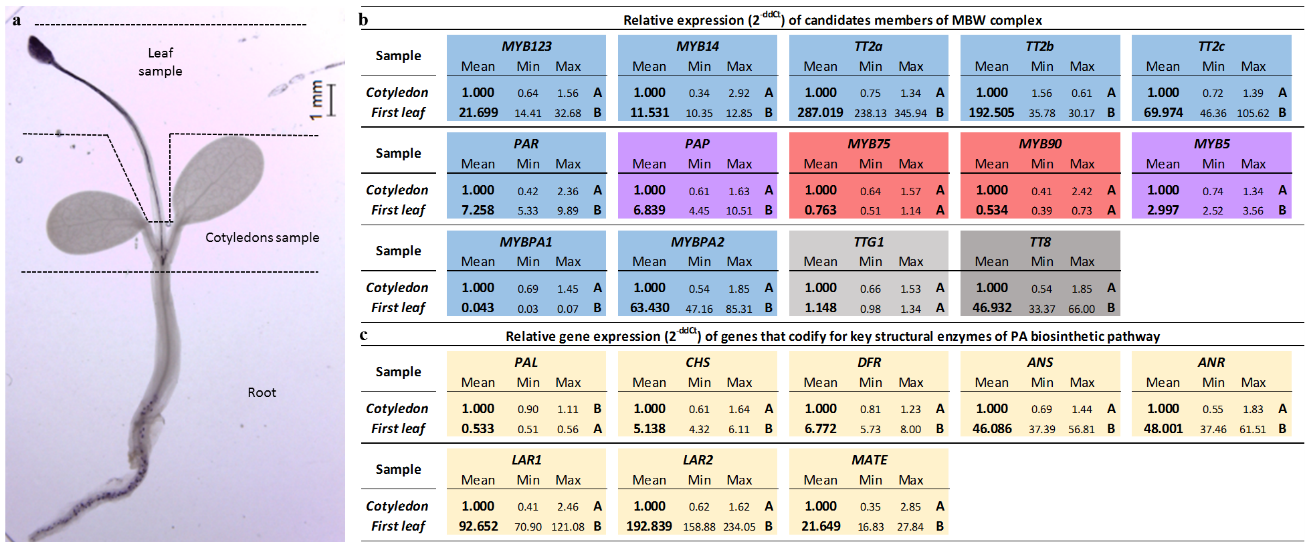


**Supplemental Figure 4.** PA and gene expression levels in *L. corniculatus* seedlings. **a** Seedling of *L. corniculatus* “charlii” destained with EtOH before staining with DMACA:HCl; blue color indicates the presence of PA. **b** and **c** Relative expression (2^-ΔΔCt^) of regulatory and structural genes of PAs, respectively. The expression level of each gene in cotyledons, arbitrarily set to 1, was used as the calibrator. Blue squares indicate MYBs belonging to subgroup A and C; purple squares indicate MYBs belonging to subgroup B and D; and red squares MYBs belonging to subgroup E. Mean values were obtained from four biological replicates; different letters among samples indicate significant difference (*P*-value <0.01, Pair Wise Fixed Reallocation Randomisation Test).
