# Supplementary material for: Multiple bHLH/MYB-based protein complexes regulate proanthocyanidin biosynthesis in the herbage of Lotus spp
Source: Planta. 2023 Dec 2;259(1):10. doi: 10.1007/s00425-023-04281-2 (PMC10693531; doi:10.1007/s00425-023-04281-2)
Supplement: Supplementary file 6 — Supplementary file6 (DOCX 110 KB) [file 425_2023_4281_MOESM6_ESM.docx]

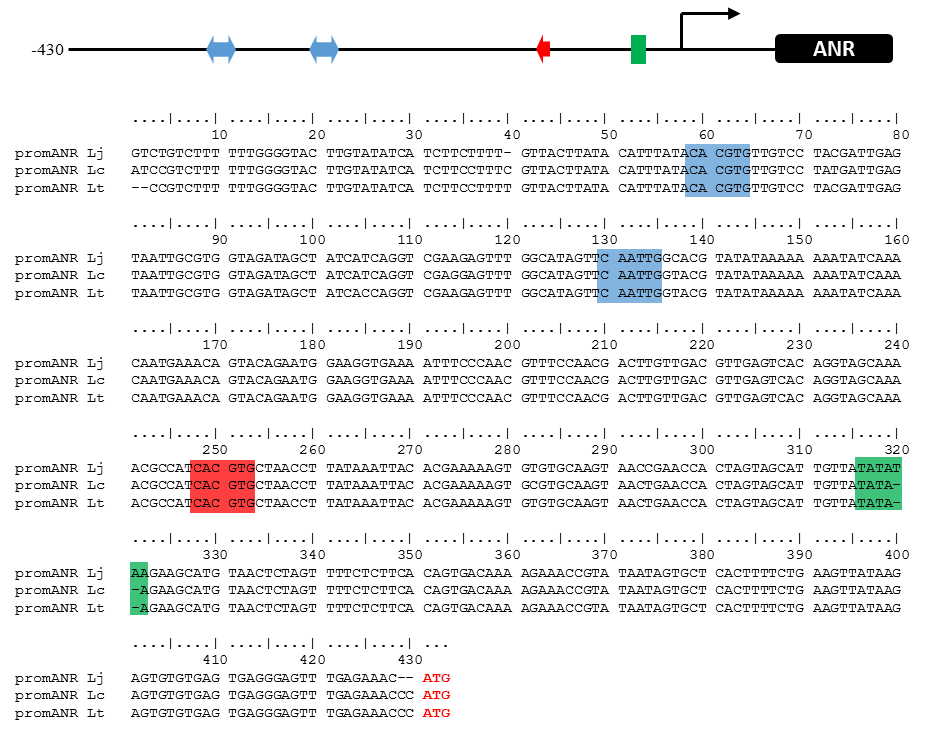


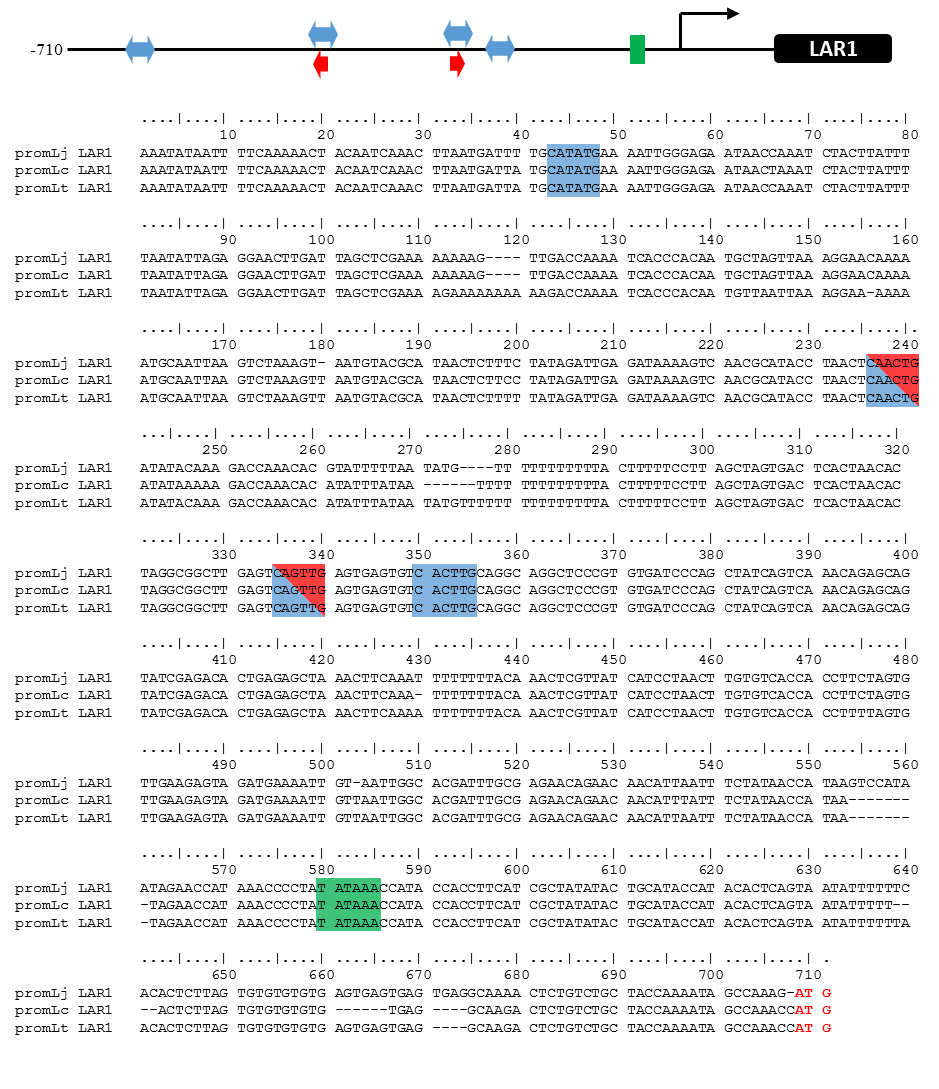


**Supplemental Figure 5.** Predicted *cis* elements (Plant DNA *cis*-element database, PLACE) in the *ANR* and *LAR1* promoter regions of *Lotus* spp. Blue arrows/squares indicate BRE motifs (CANNTG); red arrows/squares indicate MREs motifs (CNGTTR); green squares indicate TATA-box.
