# Supplementary material for: Multiple bHLH/MYB-based protein complexes regulate proanthocyanidin biosynthesis in the herbage of Lotus spp
Source: Planta. 2023 Dec 2;259(1):10. doi: 10.1007/s00425-023-04281-2 (PMC10693531; doi:10.1007/s00425-023-04281-2)
Supplement: Supplementary file 7 — Supplementary file7 (DOCX 17 KB) [file 425_2023_4281_MOESM7_ESM.docx]

**Figure S6.** Evolutionary relationships of selected R2R3-MYB proteins related to PA regulation. The evolutionary history was inferred using the Neighbor-Joining method Saitou and Nei 1987). The optimal tree with the sum of branch length = 6.38456179 is shown. The evolutionary distances were computed using the p-distance method (Nei and Kumar, 2000) and are in the units of the number of amino acid differences per site. The analysis involved 43 amino acid sequences. All positions with less than 95% site coverage were eliminated. There were a total of 388 positions in the final dataset. Evolutionary analyses were conducted in MEGA7 (Kumar et al. 2016).
