# Supplementary material for: Multiple bHLH/MYB-based protein complexes regulate proanthocyanidin biosynthesis in the herbage of Lotus spp
Source: Planta. 2023 Dec 2;259(1):10. doi: 10.1007/s00425-023-04281-2 (PMC10693531; doi:10.1007/s00425-023-04281-2)
Supplement: Supplementary file 8 — Supplementary file8 (DOCX 49 KB) [file 425_2023_4281_MOESM8_ESM.docx]

**Supplemental Table 1.** Reference sequences used to construct the phylogenetic trees.
