# Supplementary material for: Multiple bHLH/MYB-based protein complexes regulate proanthocyanidin biosynthesis in the herbage of Lotus spp
Source: Planta. 2023 Dec 2;259(1):10. doi: 10.1007/s00425-023-04281-2 (PMC10693531; doi:10.1007/s00425-023-04281-2)
Supplement: Supplementary file 9 — Supplementary file9 (DOCX 144 KB) [file 425_2023_4281_MOESM9_ESM.docx]

**Table S2** Sequences of the primers employed in this study

**Supplemental Table 2.** Sequences of the primers employed in this sy

**Gene**

**Name**

**Reference**

**qTT2aFw**

5´

CCTTCCTCAAAGTGCAGGTTTGAAA

3´

**qTT2aRv**

5´

CATCTCTGGATATATTGCCTCTCTTGATAT

3´

**qTT2bFw**

5´

GCGTTGTGGCAAAAGCTGC

3´

**qTT2bRv**

5´

TCTCTTGATACCTGGTCTTAGATAATTCAG

3´

**qTT2cFw**

5´

CAGGTTTGAAGCGTTGTGGG

3´

**qTT2cRv**

5´

ATTGCCTCTCTTGATACCTGGTTTC

3´

**qMYB123Fw**

5´

TGAACAAAGGAGCATGGACTG

3´

**qMYB123Rv**

5´

TGATATTTCCTCTTTTAATACCAGGC

3´

**qMYB14Fw**

5`

TCTCCTTGTTCAGACCAACCTC

3´

**qMYB14Rv**

5`

AGCGAAACTGCATCGTGTCC

3´

**qPARFw**

5´

TCAATAAGTGAAGCTTCATCACCACC

3´

**qPARRv**

5´

AAAAGCGTAGCAGTGCTAGTCC

3´

**qPAR2Fw**

5´

GGGCGAACAGACAATGAAAT

3´

**qPAR2Rv**

5´

GAATTCGTCCAGGTCAAAGC

3´

**qMYBPA1Fw**

5´

TTTCCCTCGGATCATCATGGTT

3´

**qMYBPA1Rv**

5´

CACCTGCAAATACTCCTCATACAG

3´

**qMYBPA2Fw**

5´

GCTACACAGAGGTCCATGGCTT

3´

**qMYBPA2Rv**

5´

ATCTCCACTGTCCTTCTCCATGATCT

3´

**qMYB5Fw**

5`

GGTGGTCTTTGATAGCAGGA

3´

**qMYB5Rv**

5`

GTGGTGGTGGAGTAGTGGAA

3´

**qPAP2Fw**

5´

GACAGAATAGTAGCAGCCAGTG

3´

**qPAP2Rv**

5´

CAGGTCCATGTCAAAGAGCAT

3´

**qMYB90Fw**

5´

GCAGCTTGAGGAAGTGGTTC

3´

**qMYB90Rv**

5´

TTCATGAGCGTCCAGTCTTG

3´

**qTT8Fw**

5´

GACAAACCCCCCGACTCGCC

3´

**qTT8Rv**

5´

CCAGGCAACCCGACACCAGG

3´

**qTTG1Fw**

5´

AACTCCGTCACCTATGACTCCC

3´

**qTTG1Rv**

5´

TCGACGCGGTTGGTGTATTCTTC

3´

**qFeEFACTf**

5´

TGACAAGCGTGTGATCGAGAGG

3´

**qFeEFACTr**

5´

GATACCTCTTTCACGCTCAGCCTT

3´

**qFePALf**

5´

GGCACCCCAATTGGTGTTT

3´

**qFePALr**

5´

CGGTGAACTGAGCAAACATGAG

3´

**qFeCHSf**

5´

CAATTTTTTGGATTGCACACCC

3´

**qFeCHSr**

5´

GAATAGGACACATGCACTTGACATG

3´

**qFeDFRf**

5´

GTCCACTTGGATGATCTTTGTCTTG

3´

**qFeDFRr**

5´

AATGTCATGGATAGTAGCCTCAGATG

3´

**qFeANSf**

5´

GGAAGTTACCAGCGACTATGCAA

3´

**qFeANSr**

5´

CCTTCCTTCTTCGAGACCCAA

3´

**qFeANRf**

5´

CCAACTCGCTACACCTGTGAATTT

3´

**qFeANRr**

5´

TGACCCGTTTAACTTTCGCCC

3´

**qFeLAR1f**

5´

GCTAAATCATTCATGGTGTTATAAACGA

3´

**qFeLAR1r**

5´

ACCTACGAGTGAAATGACAACGTCTAT

3´

**qFeLAR2f**

5´

CCTTCAGAATTTGGGCACGAT

3´

**qFeLAR2r**

5´

TTCGAACAGATGTAGGTGTATGGGA

3´

**qMATE1Fw**

5´

CCCTTACTCTCAGCTTCTCTTGG

3´

**qMATE1Rv**

5´

GAGCTTGAAATAAGGCCAAATTCC

3´

**Gene**

**Name**

**Reference**

**TT2bF**

5´

TATAACGCGTTTGGAATCACTAC + CACCATGGGAAGAAGCCCTTGTTGTT

3´

**TT2bR**

5´

AGCCGACAACCTTGATTGGAGAC + TCAATTCAGTTCACACCCATCCCA

3´

**attB1-MYBPA2**

5´

GGGGACAAGTTTGTACAAAAAAGCAGGCTTGATGGGAAGAGCTCCTTGTTGTTCTA

3´

**attB2-MYBPA2**

5´

GGGGACCACTTTGTACAAGAAAGCTGGGTTCGCTAACAACGATTCAGCAA

3´

**attB1-MYB5**

5`

GGGGACAAGTTTGTACAAAAAAGCAGGCTTAATGAGGAACCCGACAGCA

3´

**attB2-MYB5**

5`

GGGGACCACTTTGTACAAGAAAGCTGGGTTTGCTATTTGTGATGATC

3´

**attB1-TT8**

5`

GGGGACAAGTTTGTACAAAAAAGCAGGCTTGATGGTAAATCCTGTTTGGG

3´

**attB2-TT8**

5`

GGGGACCACTTTGTACAAGAAAGCTGGGTTTCAAACTAAAACATTTCTAG

3´

**SnF**

5`

TATAACGCGTTTGGAATCACTAC + CACCATGGCGCTTTCAGCTTCCCG

3´

**SnR**

5`

AGCCGACAACCTTGATTGGAGAC + TCACCGCTTCCCCCATGAAGCCTT

3´

**MYBPA1F**

5´

TATAACGCGTTTGGAATCACTAC + CACCATGGGCAGAGCACCTTGTTGTTCTAAGGTT

3´

**MYBPA1R**

5´

AGCCGACAACCTTGATTGGAGAC + TTAAATGAGTAGTGATTCGGCGAAGG

3´

**Promoter**

**Name**

**Reference**

**pANRFw**

5`

CGCGGATCCGTCTTTTTTGGGGTACTTGTA

3´

**pANRRv**

5`

CATGCCATGGGTTTCTCAAACTCCCTCACTCA

3´

**pLARFw**

5`

CGCGGATCCAAATATAATTTTCAAAAACTACAATCAAAC

3´

**pLARRv**

5`

CATGCCATGGTTTGGCTATTTTGGTAGCAGAC

3´

***TT2a***

Escaray

et al.

2017

***TT2b***

Escaray

et al.

2017

***TT2c***

Escaray

et al.

2017

***MYB123***

This work

***MYB14***

Escaray

et al.

2017

***PAR1***

This work

***PAR2***

This work

***MYBPA1***

This work

***MYBPA2***

This work

***MYB5***

This work

***MYB75***

Escaray

et al.

2017

***MYB90***

This work

Escaray

et al.

2014

***Dihydroflavonol 4-reductase***

Escaray

et al.

2014

***bHLH***

Escaray

et al.

2017

***WDR***

Escaray

et al.

2017

***Elongation factor 1-alpha***

Escaray

et al.

2014

**Primers employed in qRT-PCR assays**

**Primers used to clone and/or amplify the full length cDNA of regulatory genes**

***Leucoanthocyanidin reductase 2***

Escaray

et al.

2014

***MATE efflux family protein***

Escaray

et al.

2017

**Sequence**

***Anthocyanin synthase***

Escaray

et al.

2014

***Anthocyanidin reductase***

Escaray

et al.

2014

***Leucoanthocyanidin reductase 1***

Escaray

et al.

2014

***Phenylalanine ammonia-lyase***

Escaray

et al.

2014

***Chalcone synthase***

***LcTT8***

***Sn***

**Sequence**

This work

This work

This work

This work

***LcTT2b***

***LcMYBPA2***

***LcMYB5***

This work

**Primers used to clone and amplify the promoters of *ANR* and *LAR1* genes**

***ANR***

***LAR***

**Sequence**

This work

This work

***VvMYBPA1***

This work

I need to modify the title of each subtable
