# Supplementary material for: Multiple bHLH/MYB-based protein complexes regulate proanthocyanidin biosynthesis in the herbage of Lotus spp
Source: Planta. 2023 Dec 2;259(1):10. doi: 10.1007/s00425-023-04281-2 (PMC10693531; doi:10.1007/s00425-023-04281-2)
Supplement: Supplementary file 10 — Supplementary file10 (DOCX 18 KB) [file 425_2023_4281_MOESM10_ESM.docx]

**Table S3** PA levels in leaves of different *Lotus* genotypes. Values are mean from three biological replicates, expressed as mg PA/g of dry matter

Values are mean from three biological replicates, expressed as mg PA/g of dry matter.

Different letters represent significant differences among all the means (*P*-value < 0.05; Duncan´s test)
