# Supplementary material for: Multiple bHLH/MYB-based protein complexes regulate proanthocyanidin biosynthesis in the herbage of Lotus spp
Source: Planta. 2023 Dec 2;259(1):10. doi: 10.1007/s00425-023-04281-2 (PMC10693531; doi:10.1007/s00425-023-04281-2)
Supplement: Supplementary file 11 — Supplementary file11 (DOCX 19 KB) [file 425_2023_4281_MOESM11_ESM.docx]

**Supplemental Table 4.** Relative quantification, expressed as 2^-ΔΔCt^, of genes coding for key structural enzymes of PA pathway. The *EFACT* gene was used as reference and *L. tenuis* samples as calibrator. Means of three biological samples with the same letter are not significantly different (*P*-value ≤ 0.05, Pair Wise Fixed Reallocation Randomisation Test).
