# Supplementary material for: Multiple bHLH/MYB-based protein complexes regulate proanthocyanidin biosynthesis in the herbage of Lotus spp
Source: Planta. 2023 Dec 2;259(1):10. doi: 10.1007/s00425-023-04281-2 (PMC10693531; doi:10.1007/s00425-023-04281-2)
Supplement: Supplementary file 12 — Supplementary file12 (DOCX 38 KB) [file 425_2023_4281_MOESM12_ESM.docx]

**Supplemental Table 5.** Relative expression levels of regulatory genes in PA polymorphic *Lotus* spp. genotypes. The gene expression levels were calculated using the 2^-(ΔΔCt)^ algorithm. The expression level of each gene in *L. tenuis*, arbitrarily set to 1, was used as the calibrator. Lc C: *L. corniculatus* “charlii”, LtxLc: *L. tenuis* x *L. corniculatus*, Lc G: *L. corniculatus* “Granada”, Lc SG: *L. corniculatus* “San Gabriel”, Lb: *L. burtii*, and Lj: *L. japonicus*. Mean values were obtained from three biological replicates; different letters among samples indicate significant difference (*P*-value <0.01, Pair Wise Fixed Reallocation Randomisation Test).
